# Brain endothelial permeability, transport, and flow assessed over 10 orders of magnitude using the in situ brain perfusion technique

**DOI:** 10.1186/s12987-024-00584-y

**Published:** 2024-12-17

**Authors:** Quentin R. Smith, Haritha Mandula, Jagan Mohan R. Parepally, Jun Oki, Fancy Thomas, Helen R. Thorsheim, Abraham J. Al-Ahmad, Thomas J. Abbruscato, Per Ask, David S. Hage, Peter J. Robinson

**Affiliations:** 1 Department of Pharmaceutical Sciences, School of Pharmacy, Texas Tech University Health Sciences Center, Amarillo, TX USA; 2 National Institute on Aging, National Institutes of Health, Bethesda, MD USA; 3 Department of Biomedical Engineering, Linköping University, Linköping, Sweden; 4 Department of Chemistry, University of Nebraska-Lincoln, Lincoln, NE USA

**Keywords:** Blood brain barrier, Enhanced dissociation, Pharmaceutical screening, Diazepam, Albumin, Alpha-1 acid glycoprotein, Neurovascular unit

## Abstract

**Background:**

Cerebral blood flow normally places a limit on the magnitude of brain vascular permeability (P) that can be measured in vivo. At normal cerebral blood flow, this limit falls at the lower end of lipophilicity for most FDA-approved CNS drugs. In this study, we report on two methods that can be used to overcome this limitation and measure brain vascular permeability values that are up to ~1000 times higher using the in situ brain perfusion technique.

**Methods:**

Rat brain was perfused with physiological saline at increased flow rate and in the presence of various concentrations of plasma protein, serum albumin or alpha-acid glycoprotein. Plasma protein was added to the saline perfusion fluid to lower extraction into the measurable range using the Crone Renkin “diffusion-flow” equation to calculate brain P_o_S.

**Results:**

Cerebrovascular P_o_ was determined for 125 solutes, of which 78 showed little or no evidence of active efflux transport. Fifty of the solutes were in the lipophilicity zone (Log P_oct_ 1–5) of most FDA-approved CNS drugs. Care was taken to ensure the integrity of the brain vasculature during perfusion and to measure flow accurately using markers that had been verified for the flow rates. The results showed a linear relationship between Log P_o_ and Log P_oct_ over ~10 orders of magnitude with values for diazepam, estradiol, testosterone, and other agents that exceed prior published values by fivefold to 200-fold.

**Conclusions:**

The results show that brain vascular permeability can be measured directly in vivo for highly lipophilic solutes and the PS values obtained match reasonably with that predicted by the Crone-Renkin flow diffusion equation with care taken to validate the accuracy for the component measurements and with no need to invoke “enhanced” or “induced” dissociation.

**Supplementary Information:**

The online version contains supplementary material available at 10.1186/s12987-024-00584-y.

## Background

Brain drug delivery is determined by plasma exposure and by the transport properties of the blood–brain barrier (BBB) at the brain vascular endothelium. In the last 40 years, much has been learned regarding the critical components of BBB function, including passive diffusion as well as transporter mediated uptake and active efflux transport. More accurate methods have been developed for assessing the extent of free drug distribution in brain (e.g., K_p,uu_) as well as BBB permeability and the half time for brain drug equilibration [1–5]. For many CNS drugs, brain uptake depends not only upon BBB permeability (P) and capillary surface area (S) but also on brain blood flow (F) because CNS drugs are sufficiently lipophilic that the permeability-surface area product (PS) outpaces drug delivery from the circulation [6]. Normal cerebral blood flow ranges from 0.6 to 2.4 mL/min/g (0.01 to 0.04 mL/s/g) in rodents, depending upon anesthesia, brain region, and the state of metabolic activity of the nervous system [7–9].

To distinguish the separate contributions of F and PS, the unidirectional brain uptake transfer coefficient (K_in_), which is the primary transport parameter measured in most BBB studies, is usually analyzed using the Crone Renkin “flow diffusion” equation to correct for the separate flow- and transport-related changes in drug concentration as blood passes from the arterial to the venous end of the capillary bed [10]. At the lipophilicity levels of most CNS drugs (Log octanol partition coefficient (P_oct_) = 1–5) [11, 12], free drug extraction (E) often reaches 90–99% so that PS values exceed the measured unidirectional uptake transfer constant, K_in_, by twofold to tenfold or higher. Further, because of the exponential relationship between PS and K_in_ at these E levels, even small errors in brain F or K_in_ lead to large errors in BBB PS. In addition, over this same lipophilicity range, plasma protein binding becomes quite significant, with average free fraction (f_u_) dropping from ~1.0 to 0.01 [13]. Thus, plasma f_u_ adds an additional factor which must be incorporated into the analysis, with most studies using equilibrium dialysis for best accuracy.

In 2006, our lab published two papers showing good agreement between the Crone Renkin equation and measured BBB K_in_ at two different F rates for a series of drugs that exhibited restrictive binding to the two major Sudlow sites of human albumin, Sudlow I and II [14, 15]. Restrictive plasma protein binding is where the single pass E is less than the free fraction so that F does not limit delivery. However, a large number of rapid uptake studies have found disagreement between predicted and measured brain E with the Crone Renkin equation. In those studies, measured brain uptake exceeded that predicted using the drug f_u_ of systemic arterial circulation [16–24]. To explain these differences, researchers proposed that “enhanced"  dissociation occurs from blood proteins (primarily albumin) as blood passes from the arterial to venous end of the brain capillary bed, thus making additional drug free and available for transport [16, 18–23]. However, brain microdialysis has failed to find evidence of elevated unbound drug concentration in brain linked to the elevated plasma f_u_ [25]. The discrepancy has never been clearly resolved, as shown in two recent reviews [26, 27] where the mismatch was attributed to “non-equilibrium conditions.”

In addition, in 2009–2017, some studies reported that diazepam, the flow marker most widely used in brain perfusion field, may underestimate cerebral fluid perfusion flow by 20–100% [28–30]. One study noted that close to 1/3rd of studied CNS agents had a K_in_ greater than diazepam [30]. Unfortunately, these differences, together with the lack of agreement on “enhanced” dissociation, led some researchers to avoid using conventional BBB analyses and to instead present their data simply as brain-to-plasma concentration ratios or as simple K_in_ values, where in some cases K_in_ was taken as equivalent to PS [31–37]. Many of these values have been incorporated in literature databases, thus having impact for years beyond their initial publication.

Diazepam was first proposed as a saline perfusion fluid flow marker in 1984 [38] with a linear relation between perfusion rate and pressure up to F = 0.15 mL/s/g (i.e., 9 mL/min/g) [39, 40] with protein-free saline, and with an extraction of ~100% as measured against iodoantipyrine and labeled microspheres. Yet, diazepam also has limitations, as its lipophilicity is less than that of 35–40% of CNS drugs and it binds significantly to serum albumin, particularly to defatted human serum albumin [25, 41, 42]. Further, few studies have looked closely at flow markers for brain perfusion flow studies [43]. Thus, further work is warranted to certify flow markers and to provide a clear model that can provide reasonable PS values and flow contribution estimates.

The Crone and Renkin “flow diffusion” equation was derived to account for drug concentration differences along the length of capillaries, based upon brain PS and F [10, 44, 45],

1 $${K}_{in}=F\times E=F \left(1-{e}^{-\frac{{f}_{u}\cdot {f}_{n}\cdot {P}_{o}\cdot S}{F}}\right)$$

where K_in_ = unidirectional brain uptake transfer constant (mL/s/g) of unbound neutral drug (uptake of bound and/or charged moieties is assumed negligible by comparison); f_u_ = unbound (free) fraction (no units); f_n_ = neutral fraction in plasma or perfusion fluid (no units), which is calculated from solute pK_a_ [46]; P_o_ = cerebrovascular intrinsic membrane permeability to neutral solute (cm/s), S = vascular surface area (cm^2^/g) and F = flow (mL/s/g; we will take F = 0.024 mL/s/g or 1.4 mL/min/g for normal average F in rat cerebral cortex as our reference value in vivo) [7, 8]. Instantaneous equilibration between bound and free forms as well as ionized and neutral forms for acids and bases [44] is assumed. K_in_ can be converted into an extraction by dividing by the matched flow rate (E = K_in_/F). For acids and bases, total solute (neutral and ionized) cell permeability can be calculated as P_c_ = f_n_ × P_o_ [47], whereas for neutral solutes, f_n_ = 1 and P_o_ = P_c_. In most in vivo studies, it is difficult to measure P_o_ and S separately, and thus the parameters are usually reported together as a P_o_S or P_c_S product. When F is much greater than P_c_S, the Crone Renkin equation reduces to K_in_ = f_u_ × P_c_S or K_in_ = f_u_ × f_n_ × P_o_S, as used widely in the literature and in agreement with our 2006 studies using drugs that bind restrictively to the two main Sudlow binding sites on serum albumin [14, 15]. However, when P_c_S >> F, K_in_ approaches F and E ≈ 1.0. Under such conditions, it is difficult to derive useful P_c_S values from the Crone Renkin equation unless accurate and precise K_in_, F and f_u_ measures are available that allow one to determine PS from extractions of suitable magnitude [10, 44].

Thus, the objective of this study was to provide more meaningful brain vascular transport measures for lipophilic solutes than simple brain K_in_ or brain/perfusion fluid concentration ratios, which can be biased by flow or binding to plasma components. Methods were directed to answer the following questions: (1) How does one accurately measure BBB P_o_S of a rapid, high extraction drug, like diazepam, from protein-free saline? (2) How does one validly study BBB transport mechanism for high extraction drugs to avoid errors from flow dependence, nonspecific binding, insufficient experimental power and other factors? (3) What additional steps are necessary in experiments with plasma proteins that further complicate the analysis with differing intravascular binding kinetics (sometimes including direct measurements of protein binding kinetics, and computational simulation of these processes during passage through the brain capillary network)? and (4) What internal control components ought one include in high extraction studies to help ensure that experiments are done following the best standards?

Therefore, we sought in this paper to follow up on the discrepancies raised in past literature for lipophilic compounds with nonrestrictive plasma protein binding to determine if we could find solutions to the issues raised. We sought (a) to develop more accurate protocols for in situ brain perfusion measurements to directly determine BBB P_c_S and P_c_ for drugs in the critical lipophilicity range matching that of most current CNS drugs, and (b) to identify appropriate flow marker solutes that could be used for more accurate assessment of flow in situ in the absence and presence of protein at high flow rate. Methods have been published using differing blood pH to extend the range over which accurate PS can be measured [48, 30, 49]. We did not pursue pH differences in this study because our initial intent was for methods that could be used with all solutes, including neutral compounds, like many of the benzodiazepines. Mixed in vivo*/*in vitro methods correlate in situ K_in_ measurements with in vitro P_o_ measurements using PAMPA (parallel artificial membrane permeability assay) [29, 30]. But they do not measure BBB P_o_ directly. In several instances P_o_ could differ due to carrier-mediated uptake or efflux mechanisms. The true gold standard should be based on direct in vivo measurements.

Finally, the measurements in this paper all come from a single laboratory with multiple researchers over a number of years using common methods. This approach helps identify and control for sources of variation. For example, most all BBB K_in_ studies herein were conducted with simultaneous F and K_in_ measurements, for which we have advocated for some time for compounds that rapidly cross the BBB. In addition, we also tested whether higher flow rates and/or addition of rapidly reversible binding agents, such as bovine serum albumin [50, 51] could reduce high single pass brain extraction values sufficiently to allow P_c_S to be calculated with confidence using the Crone Renkin equation. Care was taken to avoid errors in nonspecific binding or from tracer impurities, as well as to ensure the BBB was intact with appropriate values for control PS and V_v_. In most experiments, lower affinity bovine serum albumin was used to better meet the requirements of Crone Renkin for rapid equilibrium [50, 52]. In addition, we also looked at intravascular binding and dissociation kinetics to demonstrate that they are sufficient to meet the requirements of the Crone Renkin equation. Computational model simulation studies were carried out using measured binding kinetic parameters, to ensure satisfactory compliance with the underlying assumptions of the Crone Renkin equation, specifically very rapid equilibration between bound and free forms of the drug during its passage through the brain capillaries. Comparison was performed between moderate affinity bovine serum albumin and with higher affinity, defatted human serum albumin for which accurate kinetic constants for binding and dissociation have been measured [53], We also showed that other binding proteins, such as alpha-acid glycoprotein, work as well in the experiments.

In this paper, permeability signifies the speed (cm/s) by which a molecule crosses the cerebrovascular endothelium from the blood vascular space to brain interstitial fluid. It is not assumed to confer transport mechanism, whether by protein-mediated transport or by passive diffusion. The core data set used in the bulk of the analysis (n = 78) were selected based upon knowledge that they are not strong substrates for the active efflux transporters, p-glycoprotein or breast cancer related protein. This is in alignment with reports that the great majority of FDA-approved CNS drugs do not have substantial components of active efflux transport at the BBB [54, 55]. However, the brain vascular endothelium has >50 transport carriers, of which a number have been proposed to contribute to carrier-mediated transport in and/or out of the CNS for anions, cations and neutral molecules [56]. Knowledge of transporter-mediated transport and the ability to accurately measure the magnitude of such are considered essential strengths of this project, which will be followed up in later work focused on transport mechanism and the importance of such influx to brain function.

The findings provide reasonable pathways to more accurately obtain in vivo transport data of importance in mechanistic studies of BBB transport for solutes of high E (50–100%). In addition, computational model simulation studies were carried out using measured binding kinetic parameters, to ensure satisfactory compliance with the underlying assumptions of the Crone Renkin equation, specifically very rapid equilibration between bound and free forms of the drug during its passage through the brain capillaries. (More details on this analysis will be presented in a separate paper). Recommendations are provided in the conclusion of the article to help researchers avoid pitfalls. A preliminary publication of part of this work was made in 2001 regarding brain uptake of diazepam and fatty acids [57].

## Methods

### Materials

Radiochemicals were purchased from American Radiolabeled Chemicals Inc. (St. Louis, MO), ViTrax Co. (Placentia, CA). Perkin Elmer Life Sciences (Boston, MA), and Amersham Biosciences (Piscataway, NJ). Radiotracer integrity was evaluated by reverse phase high pressure liquid chromatography (HPLC) or thin layer chromatography (TLC) using C-18 matrix.

### Animals

Male, adult, Sprague–Dawley rats (200–350 g) and CF-1 mice (30–40 g) were purchased from Charles River Laboratories (Wilmington, MA) and were allowed free access to food and water until the morning of the experiment. The experiments were performed in accordance with guidelines and protocols approved by the Institutional Animal Care and Use Committee.

### In situ rat brain perfusion

The in situ rat perfusion technique of Takasato et al. [38] was used with modification for variable flow rate [3, 39, 40, 43]. On the day of the experiment, animals were anesthetized with ketamine/xylazine (80/10 mg/kg, i.p.) or sodium pentobarbital (40–50 mg kg^−1^). Body temperature was maintained at 37 °C using a heating pad linked via rectal probe and electronic regulator.

Once surgical anesthesia was attained, an incision was made in the skin at the level of the neck and a PE-60–100 catheter filled with heparinized 0.9% NaCl (100 U/ml) was placed in the common carotid artery, after which the external carotid, pterygopalatine, and all branches of the internal carotid artery were ligated and closed shut. The preparation allowed direct delivery of perfusion fluid to the brain via the circle of Willis, which had a blood pressure of ~75 to 80 mm Hg in animals with an average arterial blood pressure of 94 ± 6 mm Hg, matching that of previous reports [58]. From this, the goal perfusion pressure was set as ~77 ± 3 mm Hg. In some experiments, the ipsilateral pterygopalatine artery (PPA) was left patent, which required a perfusion rate twice that of normal to achieve the same pressure as when the PPA was closed [39, 43].

The common carotid catheter was attached to a four-way valve linked to a thermostated (37.5 °C) syringe in a Harvard 22 dual infusion pump (Harvard Bioscience, South Natick, MA). Bicarbonate-buffered perfusion saline (128 mM NaCl, 24 mM NaHCO_3_, 4.2 mM KCl, 2.4 mM NaH_2_PO_4_, 1.5 mM CaCl_2_, 0.9 mM MgSO_4_, and 9 mM d-glucose) was equilibrated with 95% air or O_2_ and 5% CO_2_ (pH = 7.4 ± 0.05). Heparinized rat whole blood or plasma were also obtained from donor animals and used as described in Takasato et al. [38]. Radiolabeled ligands were included in perfusion fluid at concentrations of ^3^H: 1.1–5 nM and ^14^C: 0.2–0.9 µM for drugs and hormones.Tenfold higher levels were used for those agents with PS < 1×10^-3^ mL/s/g. Perfusion pressure was monitored during perfusions using a Gould strain transducer (Gould, Cleveland, OH) linked by a T connector [43, 58]. In one set of experiments, a catheter was placed in the superior sagittal sinus for determination of brain tracer single pass extraction [59].

To start the perfusion, the thoracic cavity was opened, and a rapid incision was made in the rat’s left cardiac ventricle. Then, a stopwatch was started, and a constant rate pump was activated to infuse perfusion fluid into the carotid artery feeding the brain. Lag time for the perfusion fluid to reach the brain capillaries was determined for each flow rate. In most experiments the brain was pre-perfused with tracer-free saline for 10–30 s to wash out residual plasma protein and blood elements from the cerebral blood vessels. Pump flow rate was verified weekly in separate experiments to calibrate the instrument. No in-line filters were employed, and careful steps were taken to ensure accurate determination of the tracer concentration in the perfusion reservoir and in the fluid leaving the tip of the catheter. Once preperfusion was complete, perfusion fluid was switched via a 4-way valve to matching fluid containing radiolabeled test solute and differently labeled flow tracer. Most experiments used ^14^C-labeled agent of interest with corresponding ^3^H-labeled flow or vascular volume marker. Dual tracer measurements reduced the K_in_ coefficient of variation by ≥one order of magnitude.

When perfusion was complete, the animal was decapitated, and the perfusion was stopped. The brain was dissected into regions as described by Takasato et al. [38], placed in vials and weighed. In addition, replicate samples of perfusion buffer were collected for tracer concentration determination and measurement. Tissue samples were digested at 50 °C using tissue solubilizer for 8–12 h until homogenization was complete (Packard Meriden, CT). Scintillation fluid was added, and the tracer contents were measured using dual-label liquid scintillation counting (LSC) (Beckman LS 6500, Fullerton, CA). Disintegrations per minute (dpm) were obtained from counts per minute (cpm) with automatic quench and background correction. LSC Standards were from Beckman. Care was taken to ensure appropriate tracer levels for accurate counting statistics (>10,000 counts and with ^3^H 4–5 times > ^14^C). Liquid scintillation standards were run with each experiment as positive controls. In some experiments, capillary depletion was performed [60, 61].

The unbound fraction of drug in the perfusion fluid was measured by equilibrium dialysis as previously described [14, 15], with particular care to avoid errors in nonspecific binding or tracer impurity (0.5–3%) [62]. The equilibrium time was determined for each drug of interest. The cells were suspended in a shaker maintained at 37 °C until equilibrium was achieved. Tracer integrity was confirmed by HPLC. For tracers that showed rapid degradation (e.g., chlorambucil, meta-sarcolysine, melphalan, temozolomide) ultrafiltration was performed to limit experimental time in order to maintain integrity.

In a subset of experiments, rat brain was perfused via the transcardiac method used by Thompson et al. [63]. A bulb needle (George Tiemann 160-8905) was placed through the left cardiac ventricle of anesthetized rat into the aorta. A small cut was made in the right atrium and in some cases the descending aorta was clamped. Pressure at the tip of the needle was measured with a pressure transducer connected to a chart recorder. Saline perfusion fluid with or without dextran (70 kD) or bovine serum albumin (2.7%) was perfused using a constant rate pump (Harvard Apparatus). The temperature (37 °C) and pH (7.4) of the perfusion fluid were maintained constant before and during the perfusion procedure with a heating coil linked to a controlled water bath. Perfusion fluid was aerated with 95% air/5% CO_2_. Infusion rate varied from 40 to 100 mL/min. Femoral artery blood pressure was measured just before initiation of thoracic surgery and was obtained for comparison with saline perfusion pressure.

### In situ mouse brain perfusion

Mouse brain was perfused via the external carotid artery or from the left cardiac ventricle/aorta, as previously described [39, 43]. Procedures followed that of the rat method described above, but scaled down for the smaller mouse system. The procedure was used to collect pressure values at different infusion rates and to measure BBB PS to some solutes.

### Chemistry

Radiotracer integrity was confirmed as >99% by reversed-phase high pressure liquid chromatography (HPLC) or thin layer chromatography (TLC) using C-18 supports. In some cases, tracers were repurified to achieve >99% purity. All tracers were dried under nitrogen to remove possible volatile contaminants. Several ligands, such as temozolomide, chlorambucil and iodoacetamide, are known to degrade in saline perfusate in the presence of light or at physiologic pH 7.4, so special precautions were taken to protect such solutes to minimize degradation. Further, specimens at the end of each perfusion were screened by HPLC or TLC to document tracer integrity in perfusion fluid and brain. Lipid incorporation in neutral and phospholipids was measured using LC–MS/MS or GC–MS/MS [64, 65]. For a number of compounds, BBB PS was measured by in situ perfusion using LC–MS/MS or GC–MS/MS.

### Kinetic calculations

The theory follows previous publications [3, 10, 39, 43]. Brain dpm were expressed per gram tissue and were corrected for residual intravascular tracer as C_br_ = C_tot_ − V_v_ × C_pf_ where residual vascular volume (V_v_) measured using radiolabeled inulin or sucrose.

The unidirectional uptake K_in_ into brain was calculated by two methods to attain the most accurate value. In the first approach, the initial portion of the brain uptake curve (C_br_/C_pf_ vs. net perfusion time) was analyzed by simple linear regression to calculate K_in_ as the slope of

2 $$\frac{{C}_{br}}{{C}_{pf}}= {K}_{in}\times T+ {V}_{v}$$

where T = net perfusion time. In most instances, the y intercept did not differ significantly from zero. Y-intercept accuracy was used as one index of the appropriateness of the model fit, because deflection from zero intercept could signify error from nonspecific binding.

K_in_ was also obtained by fitting a more extended time course to a two-compartment model allowing back flux, as described previously by Takasato et al. [38],

3 $${C}_{br}=({f}_{u,p}\times {V}_{u,br})\left(1-\mathit{exp}\left(-{K}_{in}\times \frac{T}{{f}_{u,p} \times {V}_{u, br}}\right)\right)$$

where f_u, p_ = unbound fraction of solute in perfusion fluid measured by equilibrium dialysis, and V_u,br_ = brain distribution volume, which is defined as the brain distribution space at steady state plateau per unbound solute concentration in perfusion fluid. In experiments using protein free saline perfusion, f_u,p_ = 1.0. The rate coefficient for brain efflux was calculated as: k_out_ = K_in_/(f_u,p_ × V_u,br_) or was measured directly in extended brain washout studies. Nonlinear regression was used to obtain the best fit K_in_ and V_u,br_ to the data (K_in_, F, f_u_, T) using the Graph Pad Prism for Windows program (version 10.3.0) (Graphpad Software, Boston MA www.graphpad.com).

Unidirectional brain extraction (E) was obtained as K_in_/F and the apparent P_c_S and intrinsic P_o_S to free drug was calculated using the Crone Renkin equation [66]:

4 $${P}_{c}S= {f}_{n}\times {P}_{o}S= -\left(\frac{F}{{f}_{u,p}}\right)ln\left(1-\frac{{K}_{in}}{F}\right)$$

For neutral molecules like diazepam, f_n_ equaled 1.00 and thus intrinsic P_o_S equaled P_c_S. For acids and bases, P_o_S was calculated as P_o_S = P_c_S/f_n_ where f_n_ = the fraction neutral of ionizable molecules (acids or bases) at the pH of the perfusion fluid (7.40); f_n_ for acids = 1/(1 + 10^(pH − pKa)^) and f_n_ for bases = 1/(1 + 10^(pKa − pH)^) [67]. Intrinsic permeability (P_o_) to neutral solute and cellular permeability (P_c,_) for total solute in cerebral cortex brain vasculature were calculated by taking the average S determined in five separate studies in rats (Average = 102 ± 8 (SEM) n = 5 cm^2^/cm^3^) [68–72]. This value was taken as 100 cm^2^/cm^3^ for simplicity in the paper and in the tables. Tissue samples were taken from across the brain and calculated variables differed from cerebral cortex as follows: 80–90% (hippocampus, caudate-putamen, and thalamus-hypothalamus), 95–105% (frontal, parietal, temporal and occipital cortex), and 110–115% (superior and inferior colliculi). These differences correlated with reported values for regional vascular surface area in rat brain. Cerebral cortex values are reported because no consistent regional differences were observed aside from those noted above based on vascular density and surface area.

The t_1/2_ for brain equilibration was calculated as [66]

5 $$Brain\,{t}_{1/2}=\frac{\mathit{ln}\left(2\right)}{{k}_{out}} =\mathit{ln}\left(2\right)/\left[\frac{{K}_{in}}{{f}_{u,p}\times {V}_{u,br}}\right]$$

where K_in_ is calculated from Eq. 1. When PS < F, Eq. 5 simplifies to

6 $$Brain\,{t}_{1/2} \approx \frac{\mathit{ln}\left(2\right)}{\left[\frac{PS}{{V}_{u,br}}\right]}$$

When f_u_ x PS >> F, Eq. 5 simplifies to

7 $$Brain\,{t}_{1/2} \approx \mathit{ln}(2)/\left[\frac{F}{({{f}_{u,p}}\cdot{V}_{u,\mathit{br}})}\right]\approx \frac{\left[\mathit{ln}\left(2\right)\times{f}_{u,p}\times {V}_{u,br}\right]}{F}$$

Some assumptions with application of the Crone Renkin equation include the following:

- Fick’s principle: Uptake rate (nmol/s) = Blood flow F (ml/s) × Arterio-venous concentration difference (C_a_ − C_v_) (nmol/ml) = C_a_ × E
- Exponential decline of the studied ligand along the length of the capillary [73–75].
- Drug uptake is not limited by radial diffusion (perpendicular to the flow) of either free or bound drug, even for those taken up very rapidly by the brain.

It is also assumed that equilibrium exists within the capillary bed between  bound and unbound as well as ionized and neutral species, and there is no effective capillary heterogeneity resulting in inefficiencies of distribution of uptake among the capillaries, so that a single capillary model can simply be scaled up to describe the whole brain or brain region.

### Statistical analysis

All data are presented as mean ± SD, unless otherwise noted. Graph Pad Prism was used for performing all the statistical analyses.

## Results

### Theory

**Fig. 1:**
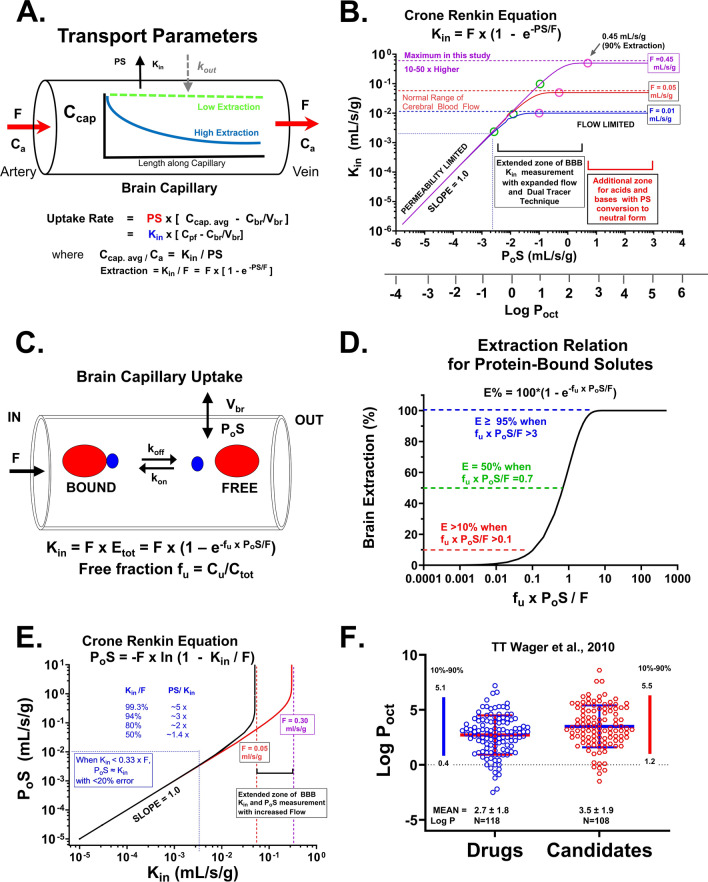
**A** Schematic diagram illustrating drug concentration gradient along the length of a brain capillary under the influence of passive diffusion and flow in the absence of protein binding. Below the diagram, kinetic equations present brain uptake using a two-compartment where C_cap_ = capillary concentration, C_br_/V_u,br_ = brain concentration divided by the brain distribution volume (V_u,br_), P = permeability, S = surface area product). Given the difficulty of measuring the concentration gradient within the capillary, most studies use the Crone-Renkin “flow diffusion equation” based upon the arterial input concentration (C_A_) and a unidirectional transfer coefficient (K_in_, mL/s/g) which predicts concentration gradient based upon the F and PS values. The unidirectional transfer coefficient (K_in_, mL/s/g) is defined as F × E = F (1 − e^−PS/F^). **B** Plot illustrating the dependence of experimental brain K_in_ based upon PS and F where f_u_ = 1. K_in_ approximately equals PS (with less than 10% error) when K_in_ < 20% of F. When K_in_ > 20% F, K_in_ depends on both PS and F until PS > 2.3 F (90% extraction), where K_in_ ≈ F with less than 10% error. The region of linear K_in_ and PS dependence can be extended by increasing F, as shown in the figure. **C** Schematic illustrating plasma protein binding in brain capillary transport with the expanded Crone Renkin equation incorporating reversible binding and free fraction of solute within the vascular compartment. **D** Plot illustrating the shape of predicted extraction relation in the presence of protein bound solute and how E shifts from <10 to >90% as [f_u_ × P_c_S/F] increases from 0.1 to 3. **E** Plot illustrating the sharp exponential dependence of P_o_S on K_in_ when K_in_ approaches F. The relationship is sufficiently strong at E > 80%, that accurate P_o_S determination is extremely difficult without simultaneous K_in_ and F measurement. **F** The strong correlation of Log P_oct_ to the 1 to 5 zone for 90% of approved CNS drugs (BLUE) and for drug candidates (RED). Data plotted is from Wager et al. [12]

Figure 1 illustrates the theory behind the two strategies used in this study to extend the zone of in vivo PS uptake measurement—(a) elevated flow (Fig. 1A, B) and (b) reduced extraction due to reversible plasma protein binding (Fig. 1C, D). The enhanced flow approach (Fig. 1A, B) was predicted to extend the linear zone of PS determination by one order of magnitude for each tenfold flow elevation. Normal cerebral blood flow in the cerebral cortex of rats and mice is ~0.024 mL/s/g) [7, 8]. The green circles in Fig. 1B illustrate the lower limit PS for each flow rate (0.01, 0.05, 0.45) for which PA ≈ K_in_ with less than 10% error. The violet circle illustrates the corresponding upper limit PS predicted at 90% extraction. At the highest flow tested, the PS limit fell in the 1–4 mL/s/g range. Figure 1C, D show the response of added plasma protein that binds ligands reversibly. Brain extraction was predicted by the Crone Renkin equation where the exponential component equaled f_n_ × f_u_ × P_o_S/F for nonelectrolytes and for ionizable solutes.

Figure 1E emphasizes the very steep exponential profile between PS and K_in_. As K_in_ approaches F, E rises to 80% (twofold multiplier), to 94% (threefold), to 99.3% (fivefold) and even higher—99.996% (tenfold). At the highest extractions, the error differences (<0.1%) are too small to determine accurately. Therefore, in this paper, we put forward alternate methods to measure E at values of K_in_, F and f_u_ that allow PS to be assayed with greater stability and accuracy.

Figure 1F shows that most (>90%) approved CNS drugs (BLUE) and drug candidates (RED) fall within Log P_oct_ 1 to 5. Hence knowledge regarding brain permeability and intravascular binding kinetics for such solutes is highly relevant.

### Identifying and eliminating nonspecific binding

**Fig. 2:**
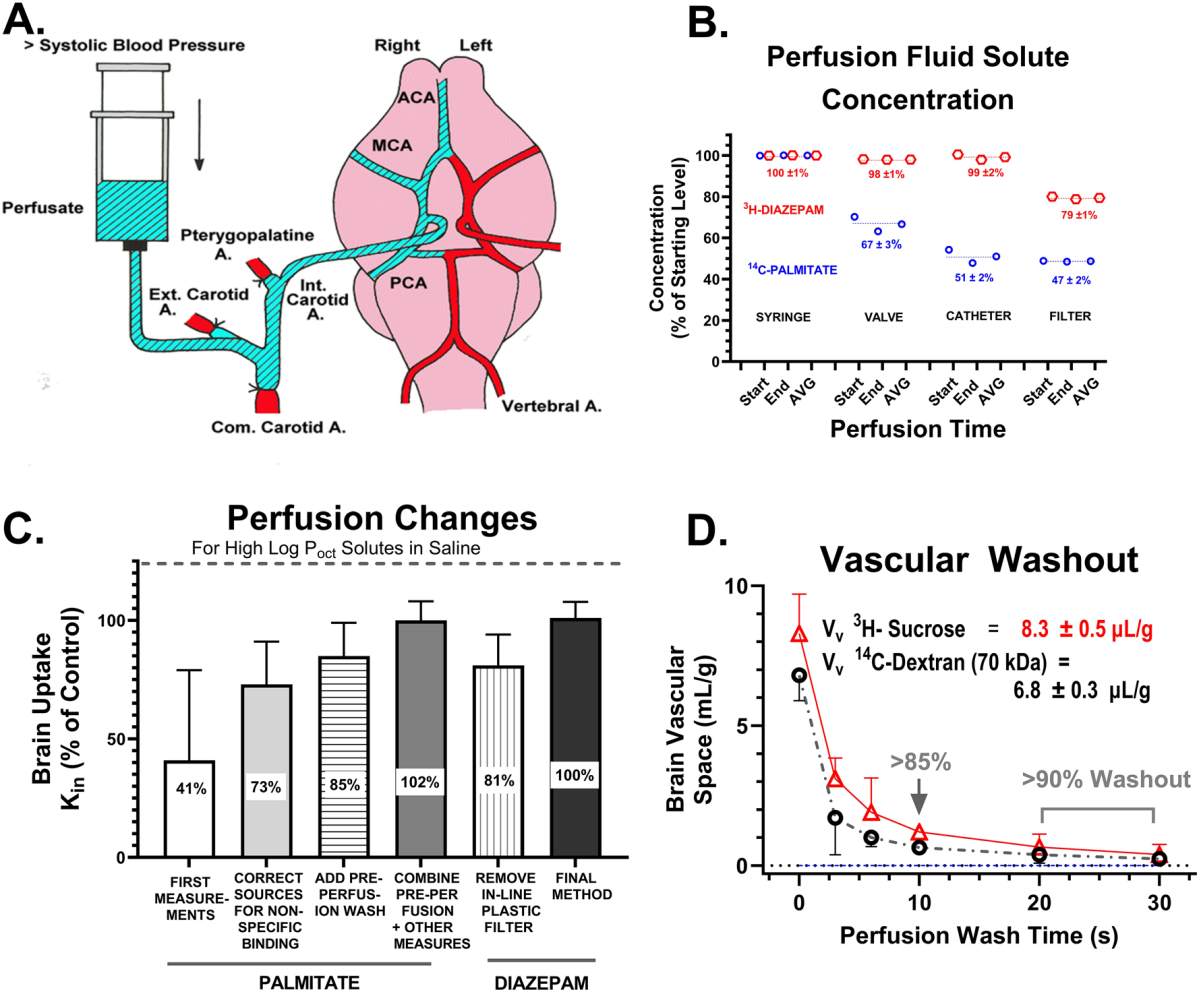
**A** Diagram illustrating the surgical preparation and blood vessels feeding the brain of the in situ brain perfusion method [3, 38, 39]. Flow rates at normal carotid artery pressure with physiologic saline are fourfold to fivefold greater at the same pressure than with whole blood, due to the fourfold to fivefold lesser viscosity of saline perfusion [93]. Shear stress remains unchanged because shear stress is proportional to the product of viscosity and flow rate [109]. **B** Plot of perfusion fluid drug concentration at different points in the perfusion apparatus, which differ due to nonspecific binding. Measurements were made in triplicate, one at the start of infusion, one in the middle, and one at the end of the perfusion. No differences were observed when albumin was present in the perfusion fluid or when the test tracer had a Log P_oct_ < 1. **C** Bar graph illustrating steps taken to identify sources of nonspecific binding and actions taken to reduce such binding by substitution of components of different material. The goal was constancy of unbound drug concentration (± 3%). **D** Time required for washout of intravascular sucrose and dextran from brain vasculature after initial 1 min loading with tracer sucrose and 70 kD dextran

Figure 2A is a schematic diagram of the in situ brain perfusion method showing the syringe source and the distribution of blood vessels that feed the brain. One immediate problem that had to be faced with high Log P_oct_ solutes was the problem of nonspecific drug binding. Such binding to perfusion pump apparatus (e.g., tubing, filters, valves, and connectors) had the potential to create significant uncertainty in perfusion fluid concentration, if appropriate steps were not taken to routinely test for such binding and to take action to reduce its impact on experimental outcomes.

Figure 2B, C illustrate several examples of binding of diazepam and palmitate at tracer concentrations and steps taken to overcome it. Palmitate with a Log P_oct_ of 5.31 showed multiple sites of binding within the perfusion apparatus which led to tracer concentration reduction of 55–70%. Diazepam, with a lower Log P_oct_ of 2.84, demonstrated only one site of significant binding (to inline perfusion filters), which reduced perfusion fluid diazepam concentration by 18–25%. Significant nonspecific binding was observed only in the absence of plasma protein and when the test solute had a Log P_oct_ of 2 or greater. Much of the binding was to valves, filters, and tubing, resulting in lower tracer concentration entering the carotid artery. Given that the exponential relation between PS and E at high E values and that many of the solutes had E values approaching 100% at normal F rates in vivo, the implications of a 20–30% lower E (due to nonspecific binding) had immense implications for calculated PS, leading to errors of threefold to 100-fold (or more). Finally, a short, 20–30 s pre-perfusion was generally performed with protein-free fluid to ensure removal of residual blood elements in brain blood vessels at the start of the experiment (Fig. 2D).

The objective in each perfusion experiment was to reduce nonspecific binding within the perfusion pump and cannula system so that the solute concentration perfusing the cerebral arteries differed by <5% from that in the main perfusion reservoir. As a double check to assess this assumption, samples were collected from the tip of the perfusion catheter at the end of the experiment to obtain final data with which to assess this assumption.

### Enhancing brain K_in_ measurement—two compartment analysis and dual tracer analysis for low coefficient of variation

**Fig. 3:**
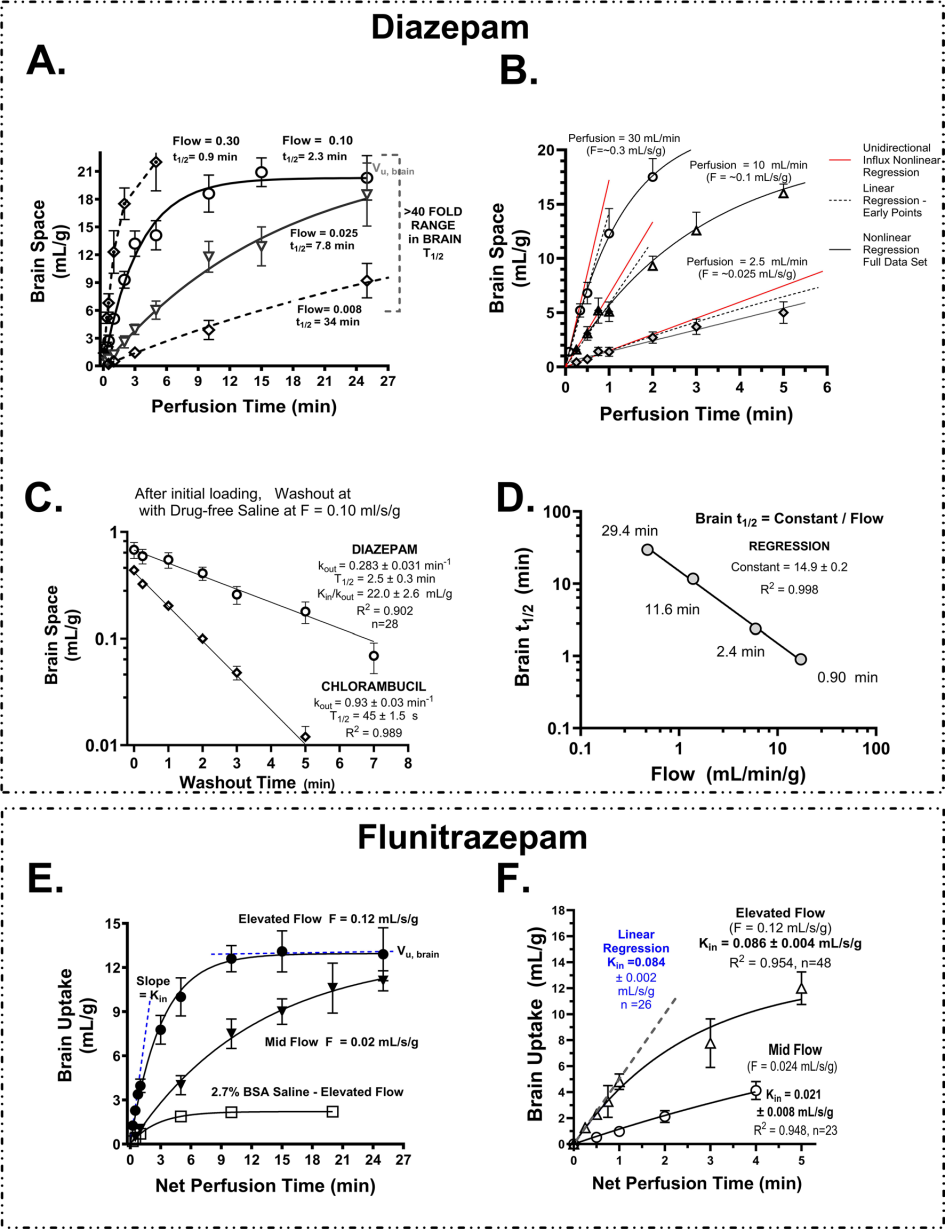
**A** Time course of diazepam uptake and equilibration in rat brain during saline perfusion in situ for up to 30 min at different flow rates and in the absence of plasma binding protein. *Solid lines* represent the best fit of the two-compartment model to the data sets at the F =  0.10 and 0.025 mL/s/g flow rates. The perfusion pump was calibrated to the desired flow rate. *Dotted lines* represent the best fits to the partial data sets at the F = 0.30 and 0.008 mL/s/g flow rates, where equilibration was <50%. **B** Initial uptake (T = 0–6 min) of diazepam into brain during saline perfusion at rates ranging from 0.025 to 0.3 mL/min. Data are presented with two compartment and linear uptake analysis. *Solid black lines* represent the best fit of the two-compartment model to the data. *Dotted black lines* represent the best fit of the linear regression equation. Solid red lines represent the predicted unidirectional uptake from the two-compartment kinetic analysis. The best fit outcomes from the regression analysis are shown in Table 1. **C** Time course of diazepam efflux from brain in situ after an initial pulse loading for 15 s followed by post perfusion wash with tracer-free fluid (F = 0.10 mL/s/g). Chlorambucil was included as a positive internal “control” for the experiment. Both plots were log linear for two orders of magnitude after the start of perfusion with tracer-free fluid. **D** Calculated half time of brain diazepam at differing flow rates. *Line* represents best fit of the two compartment model to the data for the k_out_ parameter. Plots of brain uptake and equilibration (**E**) and initial brain uptake (**F**) similar to 3A and 3B above except for flunitrazepam, which has an eightfold to tenfold lower P_oct._

**Fig. 4:**
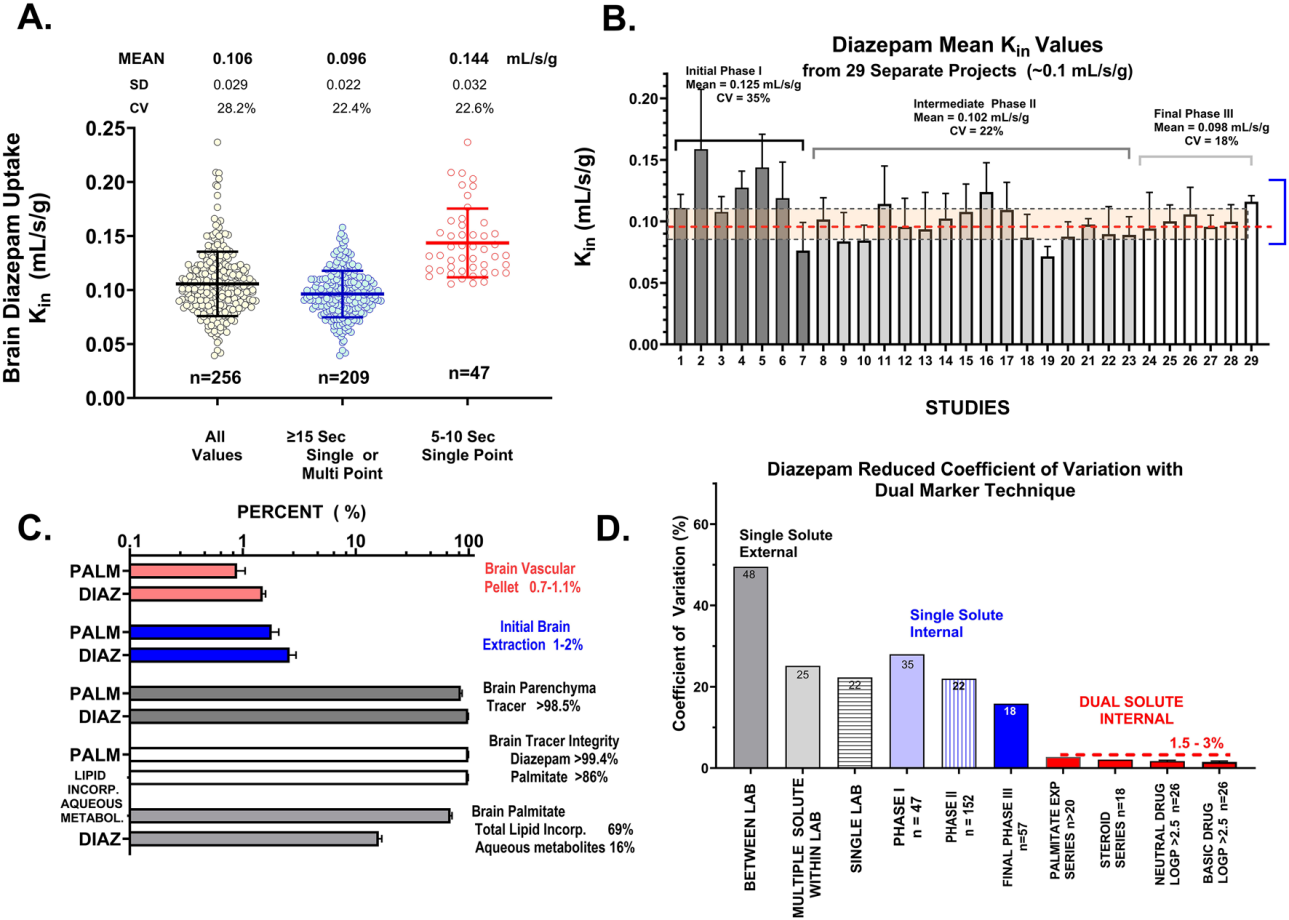
**A** Frequency distribution plot of brain diazepam K_in_ measured at a F of ~0.1 mL/s/g using buffered physiologic saline perfusion fluid (n = 256). Early measurements using very short perfusion times (5 s) were removed to obtain N = 209 that follow a normal distribution. **B** Coefficient of variation (CV(%)) of brain diazepam K_in_ across 29 different experiments with n = 11 to 21 perfusions/group. CV ranged from 16 to 29%. **C** Matching control experiments attesting to: (1) the distribution of diazepam and palmitate tracer trapped in brain vascular pellet (1%), (2) net extraction of tracer that exited brain vasculature into the subarachnoid sinus (1–2%), (3) distribution of diazepam and palmitate tracer that made it to the brain parenchymal fraction (crossed the BBB), (4) % of brain tracer which chromatographed as radioactive diazepam, (5) % of palmitate and arachidonate tracer that was incorporated into brain neutral and phospholipids or metabolized to aqueous metabolites. **D** Comparison of coefficient of variation for flow markers and high extraction solutes between single measurements (16–35%) and dual simultaneous measurements (1–3%).

Figures 3 and 4 relate to the accuracy and precision of brain diazepam K_in_ measurements, as questions have arisen regarding what was the best saline flow marker in several previous studies [28–30]. One of the core assumptions of a flow marker is that it has high extraction of ~100%. Figure 3A illustrates the time course of uptake and equilibration of diazepam at differing perfusion rates within the range of interest in this paper (F = 0.008 to F = 0.3 mL/s/g, >37-fold). The gold-standard method for BBB K_in_ determination has been the linear sloping method of Patlak et al. [76]. However, our studies found that two compartment analysis consistently gave higher brain K_in_ values than that by linear sloping for solutes with PS values ranging from 0.006 to 0.20 mL/s/g. The difference was 10–35% and likely related to inclusion of uptake values at longer time points that included significant contributions from backflux. It is hard to assess the magnitude of backflux, if one does not know the equilibrium V_u,br_ value. We have previously shown that uptake plots with the beginnings of significant backflux can appear linear but be off due to backflux that causes the y-intercept to be way too high. We have seen this repeatedly in papers. Thus, with the linear sloping method, one must not only confirm that uptake is reasonably linear but also that the y-intercept goes to a accurate value that is reasonable with the model [3]. The magnitude of the error can be quite large as shown in Fig. 3A, B and Table 1, which juxtapose results from the linear and the two compartment models for diazepam at 3–4 different flow rates. In each case, K_in_ values were equal or greater by the two compartment method than by linear analysis (Mean = 21% greater by 2 compartment model). The accuracy of the linear sloping method was confirmed using time point values which all lay in the minimal backflux zone.

**Table 1:** Regression comparison for diazepam

Perfusion rate	Analysis method	K_in_ (mL/min/g)	% Change	V_u,br_ (mL/g)	R^2^	Degrees of freedom
30 mL/min	Two-compartment model	17.2 ± 1.3	20%	22.5 ± 1.0	0.960	16
	Linear regression	14.3 ± 0.58			0.967	
10 mL/min	Linear regression Two-compartment model	6.0 ± 0.25	3%	20.3 ± 0.4	0.963	76
		5.82 ± 0.25			0.637	
2.5 mL/min	Two-compartment model	1.39 ± 0.08	39%	23.4 ± 2	0.948	63
	Linear regression	1.0 ± 0.06			0.848	
0.8 mL/min	Two-compartment model	0.48 ± 0.03	33%	20.21 ± 0.9	0.958	34
	Linear regression	0.36 ± 0.02			0.933	

Values represent mean ± SE for the number of perfusions shown

Average change in calculated K_in_ with use of 2-compartment model = 24%

The accuracy of the brain influx K_in_ value was validated by comparison to the brain efflux rate constant (k_out_) (Fig. 3C), that led to a brain distribution volume (V_u,br_ = K_in_/k_out_) = (0.101 mL/min/g)/(0.0.00472 min^−1^) = 21.4 mL/g, which agreed closely with the measured equilibration value (mean = 21.6 ± 0.8 (SEM), N = 4, n = 197 perfusions) (Table 1) and with literature values (mean 22.5 ± 1.4 (SEM), n = 6) [25, 31, 77–79]. The data support the accuracy of the K_in_ measurements with sufficient perfusion number to provide confidence limits within 30%. Brain equilibration half times varied inversely with flow over an ~30-fold range (Fig. 3D). Figure 3E, F show matching data for flunitrazepam, which exhibited comparable trends. Additional data for other lipophilic tracers are provided in Fig. 6E, F (iodoantipyrine and antipyrine).

For the plasma protein extraction method to be useful and accurate, a number of additional assumptions must be met. Figure 4A illustrates distribution plots of diazepam K_in_ at F = 10 mL/s/g. The data followed a normal distribution (n = 209 perfusions) with a single label coefficient of variation ranging from 16 to 35% over 29 separate experiments (average ~22%) (Fig. 4B). Figure 4C illustrates that only ~1% of tracer in brain was found in the vascular pellet (4C Red) at the end of the perfusion. Further, only 1–2% of the arterial concentration was found in venous perfusion fluid exiting the superior sagittal sinus, indicating extraction of 98–99% (Fig. 4C Blue). Over 98.5% of brain ^14^C-diazepam tracer chromatographed as intact diazepam by HPLC (Fig. 4C, Dark Gray and White). Similarly, brain ^14^C -palmitate and ^3^H-arachidonate were 69 and 90% intact and had been incorporated into brain phospho- and neutral-lipids within 60 s in the brain parenchymal fraction, after capillary depletion (Fig. 4C Light Gray). Together, the results provide support that the brain diazepam and palmitate K_in_ values represented tracer that was primarily intact and had crossed the BBB*.*

Finally, methods were tested to identify techniques that reduced the coefficient of variation of brain uptake K_in_ and F measurements, which equalled E when put together as the K_in_/F ratio. Very early in the analysis, dual tracer measurement of flow and brain uptake K_in_ in the same perfusion was found to reduce the linked coefficient of variation measurement between the two parameters by >tenfold (Fig. 4D). The coefficient of variation of single label flow measurement, in our laboratory as well as others, was commonly in the 15–25% range, whereas that related to the difference in F and K_in_ was tenfold lower (1.5–3%) when both were measured simultaneously in the same animal. Reduced variance was found (Fig. 4D) in four different tracer comparisons: diazepam vs. palmitate (n > 20), diazepam vs. steroid hormone (n = 18), diazepam vs. other benzodiazepine (Log P_oct_ > 2.5) (n = 26), and diazepam vs. lipophilic basic drug (Log P_oct_ > 2.5) (n = 26), for a total of 106 separate dual tracer measurements. The single solute data in Fig. 4D for our laboratory are the 256 perfusions which constitute Fig. 4A, B. The three blue bars in Fig. 4D represent mean data from each of the Phase 1, 2 and 3 groups in Fig. 4B. Given the striking reduction in variation, flow tracers were routinely included in subsequent uptake K_in_ experiments for solutes having a brain K_in_ within one order of magnitude of brain F.

### High flow K_in_ results and validation—diazepam

**Fig. 5:**
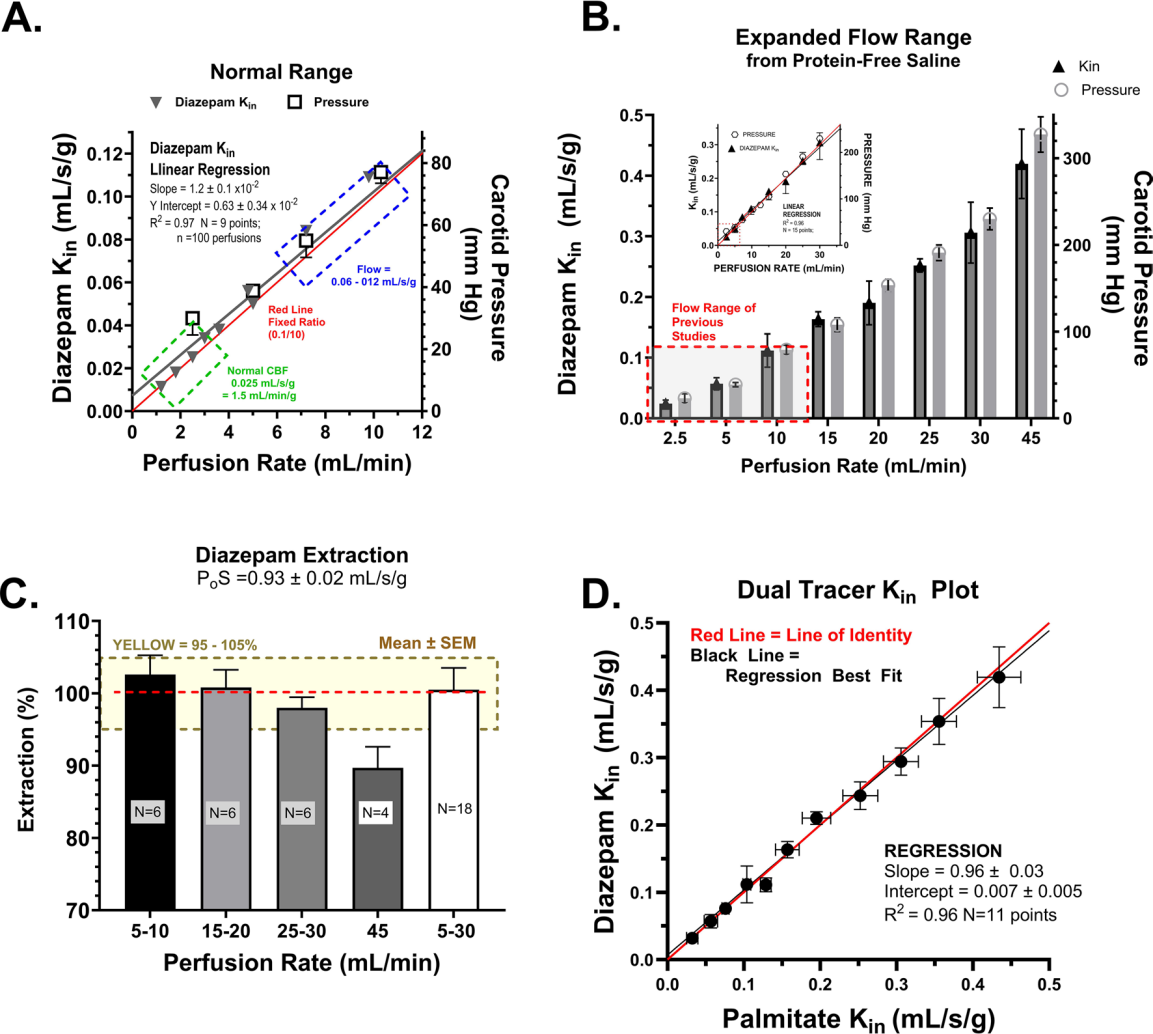
**A** Relation of measured brain diazepam K_in_ (Left Y-Axis) and carotid artery perfusion pressure (Right Y-Axis) to perfusion rate over the flow range of most published in situ brain perfusion methods (1–10 mL/s/g) where the pterygopalatine artery is ligated and the heart is stopped just prior to the start of the infusion sequence. The *solid lines* represent the best fit of the linear equation to the data (*Black* Pressure vs. Pump perfusion rate and *Red* Cerebral perfusion fluid flow vs. Pump perfusion rate). The *box outlined* *in* *blue* delineates diazepam K_in_ values from 50 to 100% of the original Takasato et al., brain flow rate (0.01 mL/s/g). Such values overlap with pressure values of 40–80 mm Hg (saline perfusion fluid). The *box in* *green outlines* diazepam K_in_ and carotid perfusion pressure at flow rates used in a good number of published studies, where the arterial perfusion pressure (5–20 mm Hg) equals or is less than published values for critical closing pressure (~20–30 mm Hg) and tissue pressure (6–7 mm Hg) [120–122]. **B** Diazepam K_in_ plotted vs. pump perfusion rate (mL/min) at brain perfusion F rates matching those in Fig. 3A and going above to F ~ 0.5 mL/s/g (maximal) and 45 mL/min perfusion rate. The *small inset* panel illustrates the linear regression of the data. **C** Brain extraction (E = K_in_/F) for diazepam at differing perfusion flow rates up to 45 mL/min. Extraction fell within ±5% of 100% up to a perfusion rate of 30 mL/min. The *yellow box* outlines the area of 95–105% extraction. **D** Plot of measured brain K_in_ to diazepam vs. K_in_ to palmitate measured simultaneously in the same animals. Values represent means ± SEM for n = 4–20 perfusions. *Lines* in Plots A, B and D are the linear regression best fits of the model equation to the data. *Red line* = Line of Identity. This comparison plot of measured flows is widely used in the cerebral blood flow literature to identify flow markers that are not sufficiently permeable to maintain the line of identity.

The high flow rate method required validation of flow markers with brain single pass extraction of >90% over a range of flow values. Figure 5A, B present brain diazepam K_in_ and carotid perfusion pressure as a function of carotid perfusion pump flow up to an infusion rate of 12 mL/min (A) and 45 mL/min (B). In both plots, pressure and brain flow rate rose linearly with pump perfusion rate over the tested range. Carotid pressure at 30 mL/min infusion rate reached a pressure value (200–240 mm Hg), just into the range previously identified for hypertensive BBB damage [58]. Therefore, flow was not increased above 50 mL/min.

Brain diazepam extraction values calculated from the data are shown in Fig. 5C. In each perfusion, radiolabeled palmitate was included in the perfusion medium as a flow marker along with labeled diazepam. The single pass diazepam extraction equals (K_in_ Diazepam)/(K_in_ Palmitate). Perfusion fluid was protein-free physiologic saline and included both a prewash (20 s) and a postwash to remove any tracer that had adsorbed to the vascular wall. Spector, Ouellet, and our own lab have previously shown that the single pass brain extraction of palmitate is 100% in the absence of plasma protein [57, 80, 81]. In this set of experiments with differing flow rates, brain extraction held firmly to ~100% except at the highest flow rate (45 mL/min) where the mean diazepam E was ~90% (Fig. 5C). Overall, the results confirm that diazepam readily penetrates the BBB and provides near complete brain extraction from protein-free saline up to flow rates of 0.3 mL/s/g.

The relation between the diazepam and palmitate K_in_ (Fig. 5D) was linear across the measured range. Based upon this analysis, the diazepam K_in_ from saline perfusion fluid is at least 0.40–0.45 mL/s/g, or ~10 to 20 times greater than previous estimates by other groups (0.015–0.06 mL/s/g). Further, given that the brain extraction at the highest flow rate was 90% at F = 0.45 mL/s/g, the Crone Renkin equation predicts that the minimal P_o_S is ~2.3 times K_in_ or = 0.41 × 2.3 = 0.94 mL/s/g. This is ~15 to 60 times greater than prior BBB diazepam PS estimates (PS prior = 0.030–0.060 mL/s/g). Thus, the results provide a new lower limit for diazepam PS at the BBB: 0.94 mL/s/g.

**Fig. 6:**
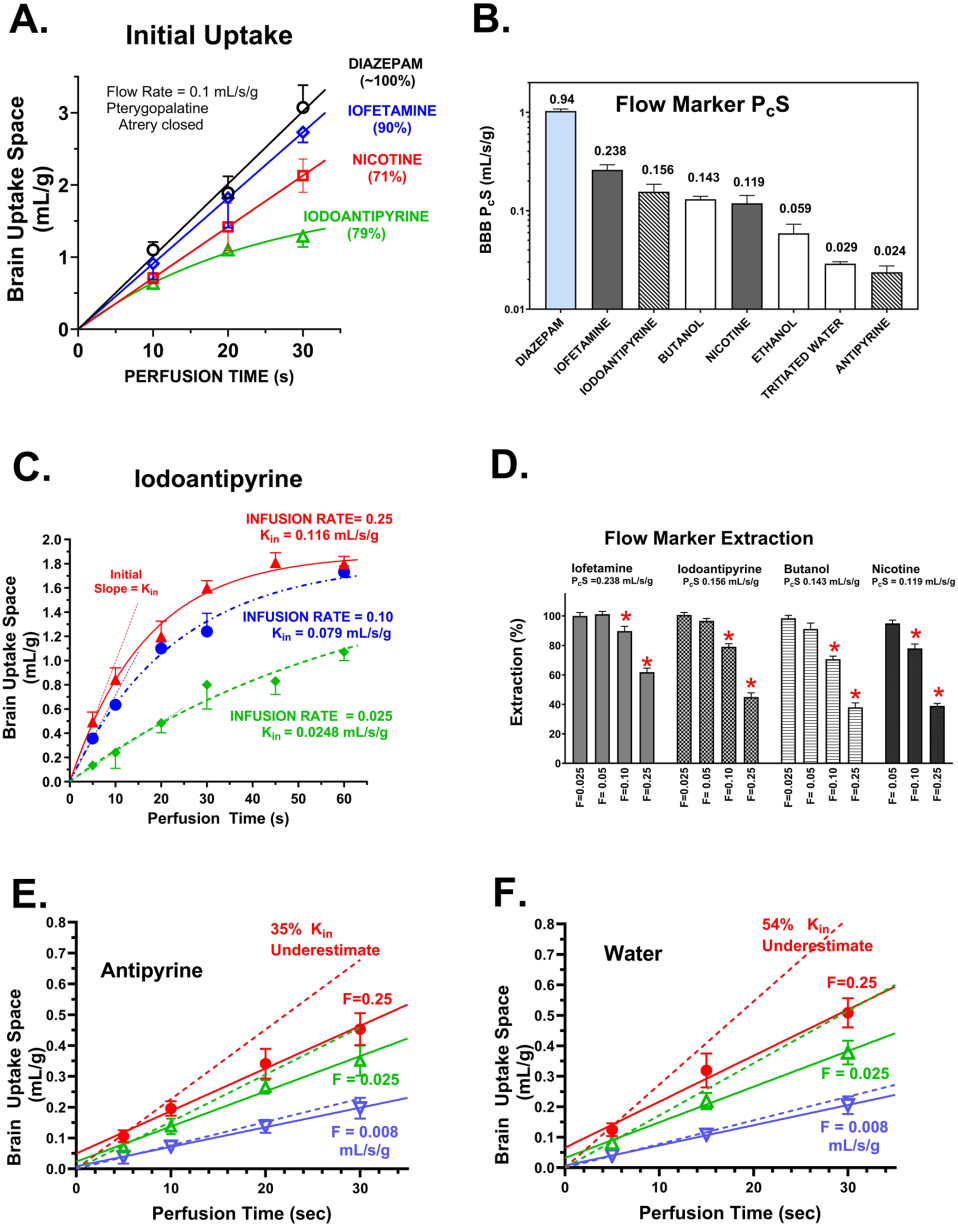
**A** Time course for initial brain uptake of diazepam, isopropyl-iodoamphetamine (iofetamine), nicotine and iodoantipyrine from protein-free physiologic perfusion fluid. **B** Measured intrinsic P_c_S values for eight flow markers. The calculated mean P_c_S value for each solute is shown above its bar value. The maximal in vivo flow rates predicted to give 95 and 80% extraction can be calculated for each flow marker by dividing the f_u, pf_ × P_c_S by 3 and 1.8 respectively. **C** Time course of brain equilibration of iodoantipyrine at differing flow rates using protein-free physiological perfusion fluid. *Lines* represent best fit of the two compartment model to the data. **D** Brain extraction values for four markers at 3–4 differing flow values. **E**, **F** Time course for antipyrine (**A**) and water (**B**) uptake from saline perfusion fluid at differing perfusion flow rates. Uptake space varies for both compounds at different flow rates. *Solid lines* are best fit linear regression values, whereas dotted lines show predicted unidirectional uptake calculated from the two compartment model. Values of bars are means ± SEM

To further test the assay, diazepam uptake was measured simultaneously to that of seven traditional cerebral blood flow markers from the neurophysiology and neuroscience fields. This included isopropyl-iodoamphetamine (also called iofetamine), iodoantipyrine, butanol, nicotine, ethanol, water, and antipyrine (Fig. 6). As illustrated in Fig. 6A, the time course of brain uptake varied significantly among markers. Iofetamine came closest to approximating diazepam. Best fit BBB P_c_S values equaled 0.238, 0.156, 0.143, and 0.119 mL/s/g for iofetamine, iodoantipyrine, butanol and nicotine, respectively (Fig. 6D). The two-compartment method to calculate K_in_ was particularly valuable with low V_u,br_ compounds like iodoantipyrine (Fig. 6A), antipyrine (Fig. 6E), and water (Fig. 6F), where tracers completed the linear zone within 10–20 s at the highest flow rates and reached near equilibration in several instances by 60–90 s. Peak flow rates for 90% E were calculated from the Crone Renkin equation and equaled 0.38 (diazepam), 0.13 (isopropyl-iodoamphetamine), 0.068 (iodoantipyrine), 0.064 (butanol), and 0.052 (nicotine) mL/s/g for protein-free physiologic saline (pH 7.4). Matching values in mL/min/g are 22.7, 7.6, 4.1, 3.8, and 3.1, respectively.

### Barrier integrity, vascular volume, and capillary surface area at different flow rates

**Fig. 7:**
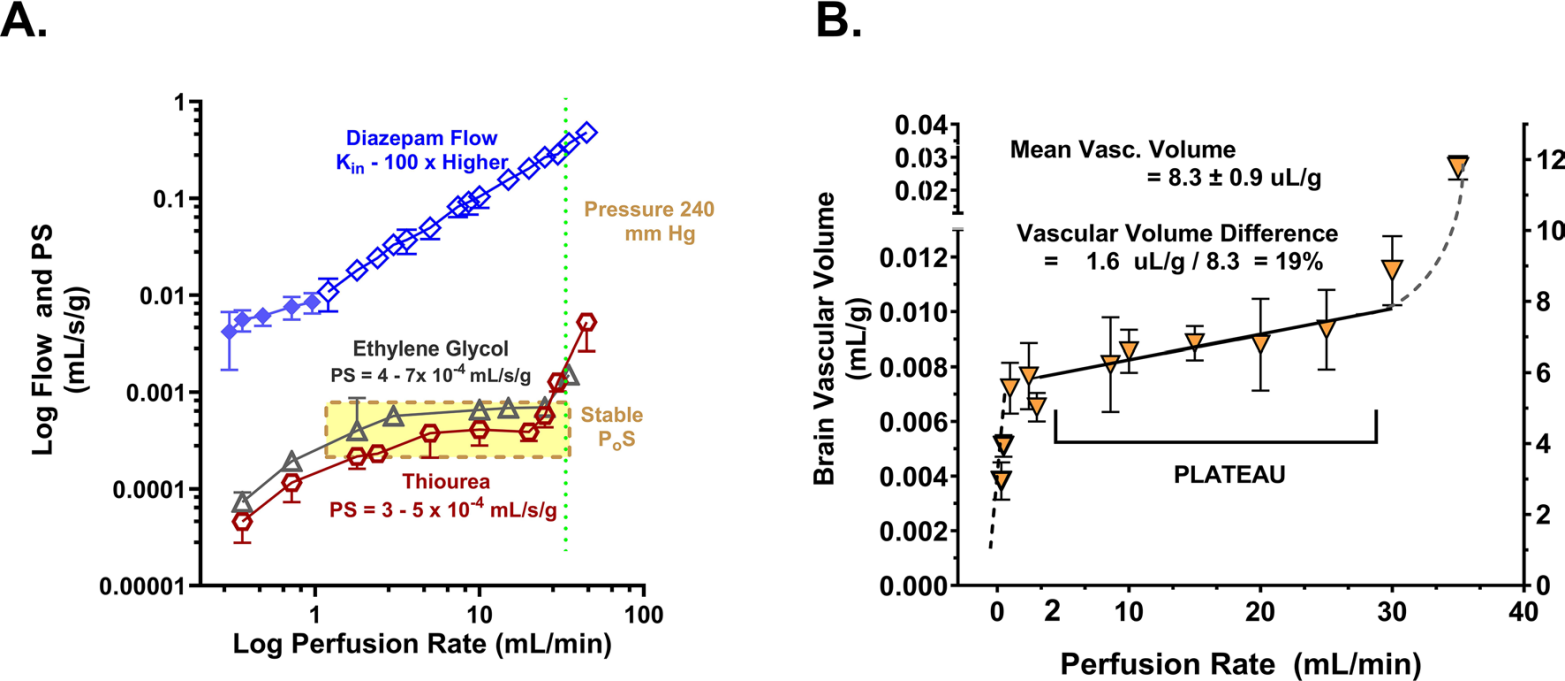
Brain P_o_S (**A**) as well as vascular volume (V_v_, mL/g) (**B**) measured at a series of flow values ranging from <1 to 45 mL/min with the in situ brain perfusion technique using protein-free saline perfusion fluid. BBB integrity was assessed using two small, polar solutes—thiourea and ethylene glycol which exhibited BBB PS values of ~10^–3^ mL/s/g. Both molecules are neutral nonelectrolytes, and thus P_c_S = P_o_S. For both compounds, BBB P_c_S remained stable from 2 to 25 mL/min. At perfusion rates <1–2 ml, PS declined steeply. At perfusion rates >30 mL/min pressure exceeded that associated with hypertensive BBB damage (>200–240 mm Hg) and was accompanied by significantly higher PS values to both tracers. However, diazepam K_in_ increased linearly through that hypertensive region with no sign of BBB damage deflection. A similar pattern was observed in inulin vascular volume (**B**)

Figure 7A presents flow-corrected P_o_S values for two passive permeability markers, ethylene glycol, and thiourea, with normal P_o_S values 3–6 of 10^–4^ mL/s/g over the bulk of the flow range, 1–25 mL/min. P_o_S values declined markedly at perfusion pump flow rates less than 1 ml/min and rose sharply at pump flow rates >30 mL/min. The marked P_o_S rise at the 30 mL/min saline perfusion rate matched in pressure the threshold (200–240 mm Hg) previously determined for hypertensive barrier damage [58, 82, 83]. The intermediate rate zone of 2–30 mL/s/g provided a plateau over which BBB P_o_S was successfully maintained as approximately constant.

Brain V_v_, shown in Fig. 7B for inulin, followed a similar pattern, as anticipated by greater leakage of the vascular tracer following barrier damage. The results show that flow rates of 1–25 mL/min are associated with stable integrity of the BBB for lipophilic drug P_o_S determination. Further, the two orders of magnitude difference in K_in_ values between the lipophilic diazepam and the polar markers (ethylene glycol and thiourea) at the highest pump rate (45–50 mL/min) lends support to the concept that transcellular P_o_S for lipophilic solutes may be accurate well into the hypertensive zone (Fig. 7A). The same trends were shown for V_v_ in Fig. 7B. On the opposite end of vascular pressure, low flow rates with low viscosity fluid such as saline can lead to incomplete vascular perfusion and closure of vessels, as discussed later, and should be avoided.

**Fig. 8:**
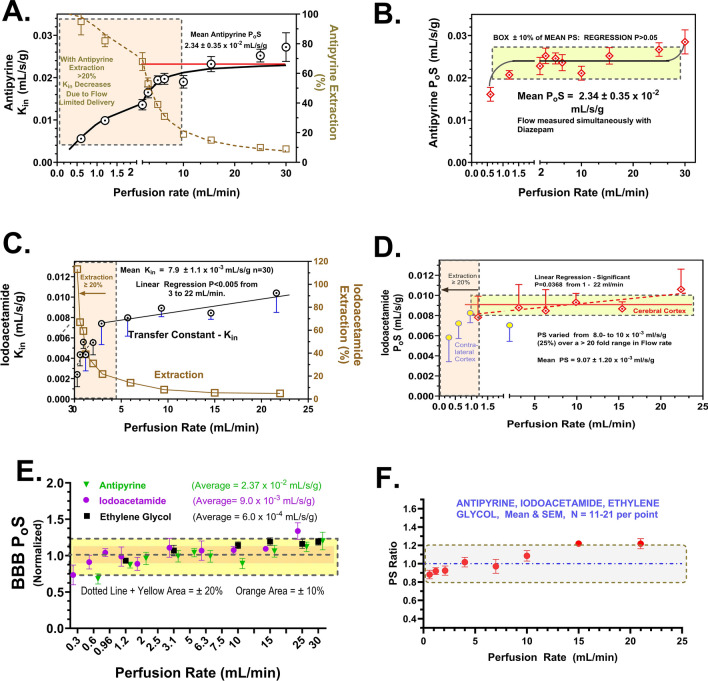
Plots of measured brain K_in_ and E (**A**, **C**) as well as BBB PS vs. perfusion rate for antipyrine (**A**, **B**) and iodoacetamide (**C**, **D**). For both compounds, E decreased and K_in_ rose with increasing flow rate, as predicted by the Crone Renkin equation. BBB PS values for both solutes did not differ over the perfusion rate of 2–25 mL/min. At perfusion rates <1–2 ml, PS declined significantly, as was noted in Fig. 7. **E**, **F** Combined plot of normalized PS values for three passive permeability markers, showing that all but one value fell within ±20% of the mean

As the first step in testing the developed method, brain K_in_, E and P_o_S were determined at differing flow rates for three moderate permeability markers—antipyrine, iodoacetamide and water, which have been widely assessed in man and animals. Figure 8A, C show brain K_in_ and E data for antipyrine and iodoacetamide which varied significantly with flow, as anticipated. In contrast, brain P_o_S values for both tracers (Fig. 8B, D) remained stable within ±20% over the flow range at values that matched well those published for the in vivo literature [8, 84–89]. Normalized P_o_S values were combined from three of the tracers (Fig. 8E, F), which showed ±16% variability and matched that (18%) reported for capillary diameter and surface area changes reported by Duelli and Kuschinsky [90] over a comparable flow range in vivo. The results emphasize the importance of measuring flow-adjusted P_o_S values instead of K_in_ because K_in_ changes with flow.

### PS values for high lipophilicity solutes by elevated flow

**Fig. 9:**
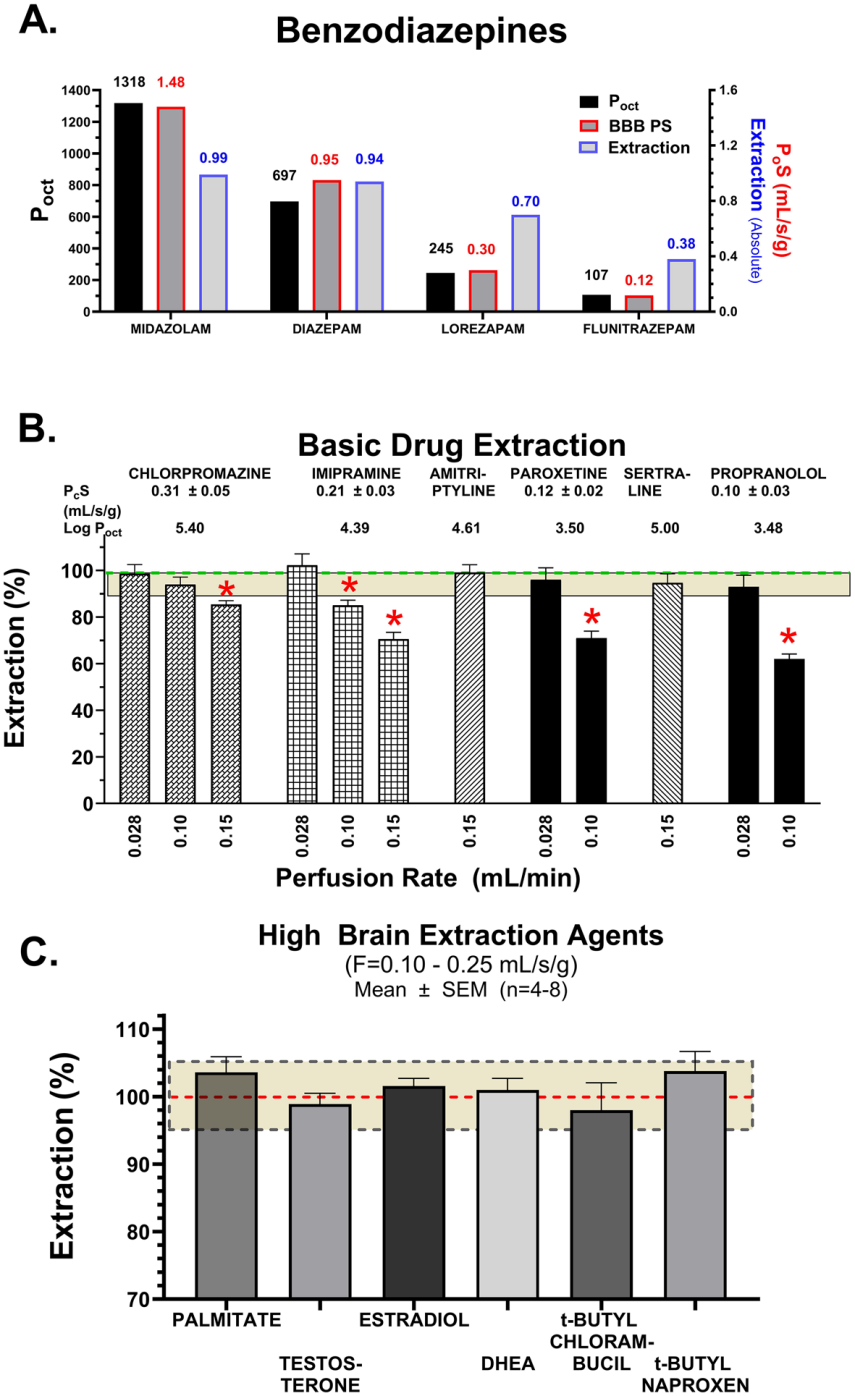
Brain extraction values for four benzodiazepines (**A**), six lipophilic basic CNS drugs (**B**) and six high extraction agents (**C**) determined at differing saline flow rates, using the elevated flow perfusion method to measure high permeability at the BBB. Brain perfusion flow rate varied over a 10-fold range from F = 0.025 to 0.25 mL/s/g.  At higher F values (>0.25 mL/s/g) BBB permeability integrity begins to erode from hypertensive damage.  Thus, the method may be limited to BBB P_o_S < 5-20 mL/s/g.

Figure 9 illustrates use of the elevated flow technique to measure brain uptake for high extraction compounds. Figure 9A shows P_oct_, BBB P_o_S, and E for 4 benzodiazepines. P_o_S correlated well with lipophilicity for all 4 compounds over an order of magnitude range, with midazolam having the highest permeability. In contrast, E varied to a more limited degree, from 70 to 99% for 3 of the compounds used in the treatment of status epilepticus and by 38% for flunitrazepam. Figure 9B shows extractions and P_c_S values for a series of lipophilic basic drugs, that have been suggested to have greater BBB permeability than diazepam. Four of the six basic drugs exhibited extractions that declined with elevated flow rate, indicating a P_c_S value less than diazepam, which was used as the flow tracer. Calculated P_c_S values equaled 0.31 ±  0.05, 0.21 ± 0.03, 0.12 ± 0.02, 0.10 ± 0.03 for chlorpromazine, imipramine, paroxetine, and propranolol, respectively that exhibit flow dependent K_in_ values <100%. For amitriptyline and sertraline as well as the 6 solutes in Fig. 6C, E was 100% to the highest flow tested. Therefore, for these 8 solutes, additional methods were needed to measure BBB P_c_S.

### Serum albumin

**Fig. 10:**
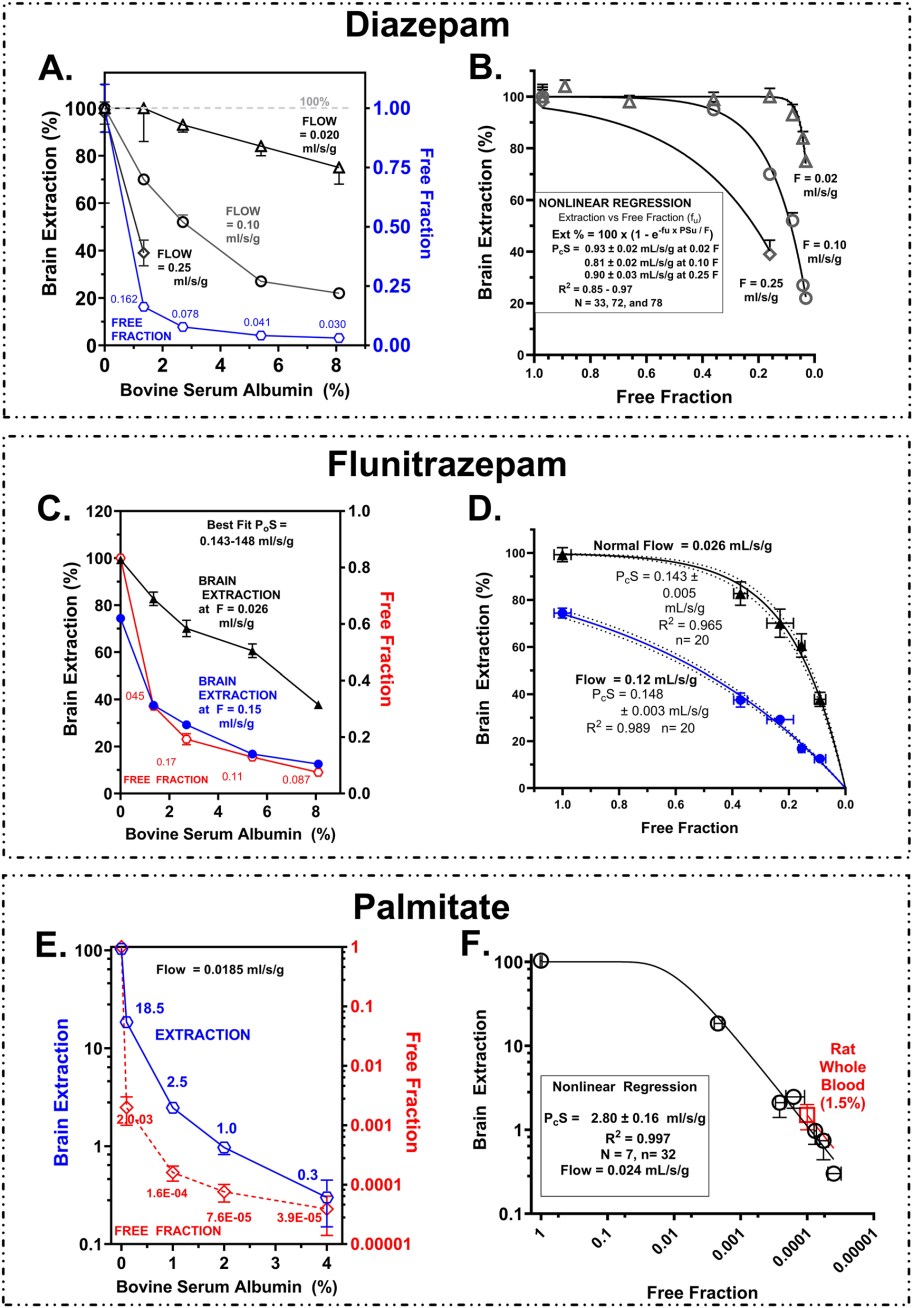
**A**, **C**, **E** Plots of brain extraction for diazepam, flunitrazepam and palmitate as a function of added bovine serum albumin to the saline perfusion fluid. Free fractions were measured by equilibrium dialysis. Lines in the plots connect the points. **B**, **D**, **F** Plots of brain extraction vs. free fraction where the *line* represents the best fit of the Crone Renkin equation to the data to provide predicted BBB P_c_S values. Plot **F** also shows data from one series of perfusions using rat whole blood at an infusion adjusted for the difference in viscosity between the fluids.

Addition of plasma protein is another approach that can be used to bring perfusion fluid or plasma f_u_ into the range where P_c_S can be calculated from E using the Crone Renkin equation (Fig. 1D). Figure 10 illustrates extraction values measured for diazepam, flunitrazepam and palmitate at differing perfusion fluid concentrations of bovine serum albumin. Bovine serum albumin was specifically chosen because it has lower binding affinity and more rapid drug exchange than human serum albumin [52]. The results in Fig. 10A, C and E were graphed as E vs. f_u_ and the Crone Renkin equation, modified for plasma protein binding, was fit to the data (Fig. 10B, D, F) using nonlinear regression to calculate the P_c_S (R^2^ > 0.95). Best fit P_c_S values equaled 0.81–0.93 (diazepam − neutral), 0.14 (flunitrazepam − neutral), and 2.8 for palmitate. For the neutral compounds, P_c_S’s are equivalent to the intrinsic values (P_o_S). For palmitate, adjustment for the neutral fraction (pH 7.4) makes the P_o_S = 1,254 mL/s/g. All five P_o_S values far exceed previous estimates. The BBB P_o_S for diazepam from four separate experiments: 0.94- 0.95 (variable F), 0.93 (variable f_u_ at 0.024 F), 0.81 (variable f_u_ at 0.1 F), and 0.91 (variable f_u_ at 0.25 F), compiles to an overall mean P_o_S of 0.903 ± 0.031 (SEM) mL/s/g.

**Fig. 11:**
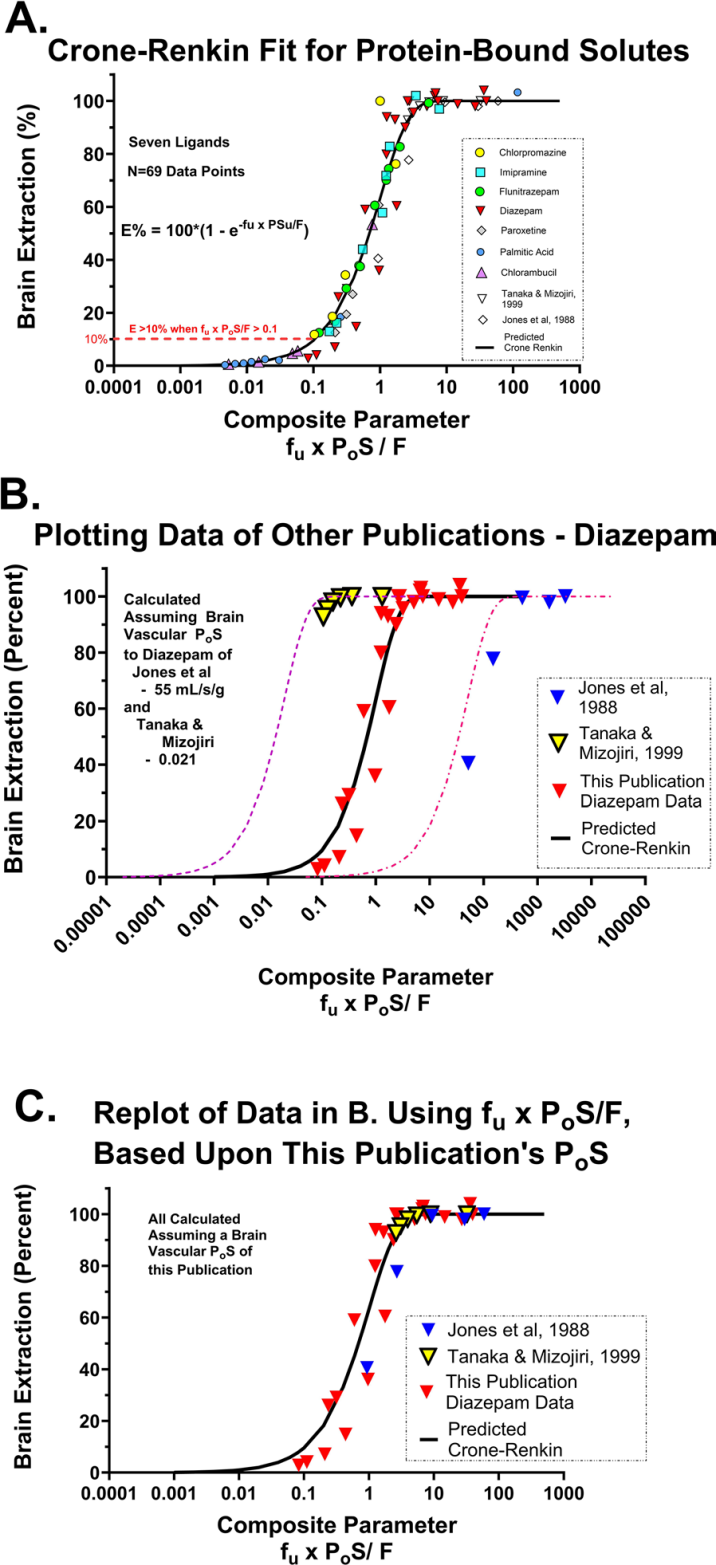
**A** Plot of measured brain extraction with the in situ brain perfusion method vs. f_u_ × P_o_S/F. Data are shown for seven solutes in **A** with the *line* representing the Crone Renkin equation. **B** Data extracted from publications by Jones et al. [20] and Tanaka and Mizojiri [22] showing deviation from the Crone Renkin model using the measured parameters. **C** Representation of the same measured extraction data replotted via f_u_ × PS/F using the PS derived in this study

To evaluate the validity of the approach, extraction (E = K_in_/F) values for seven solutes at differing flow rates and free fractions were plotted against −f_u_ × P_c_S/F of the Crone Renkin equation to determine how well the data held to the equation. Results matched well brain extraction over five orders of magnitude (Fig. 11A). This was taken as solid evidence in support of the approach to measure P_c_S to highly lipophilic agents.

Figure 11B plots data extracted from publications by Jones et al. [20] and Tanaka and Mizojiri [22] on the effect of albumin binding to diazepam and its impact on BBB uptake. The fit of the obtained data to the Crone Renkin plot differs by 1–2 orders of magnitude based upon their diazepam P_o_S values. However, when plotted using the diazepam P_o_S determined in the present study, the data matched well that expected for the CR equation (Fig. 11C). We hypothesize that the difference leading to poor quality of fit using the Crone Renkin equation with previous data was not due to difference in f_u_, as originally suggested, but because researchers had significantly underestimated P_o_S values for control BBB uptake. For example, the traditional BBB PS for diazepam required ~twentyfold greater f_u_ values to fit the data. However, the differences resolve with typical values of f_u_ when the new P_o_S value (0.90 mL/s/g) is substituted. The same held for seven other ligands. The origin of the solution arises from the exponential term in the Crone Renkin equation where PS and f_u_ appear as a joint product - f_u_ x P_o_S/F.

**Fig. 12:**
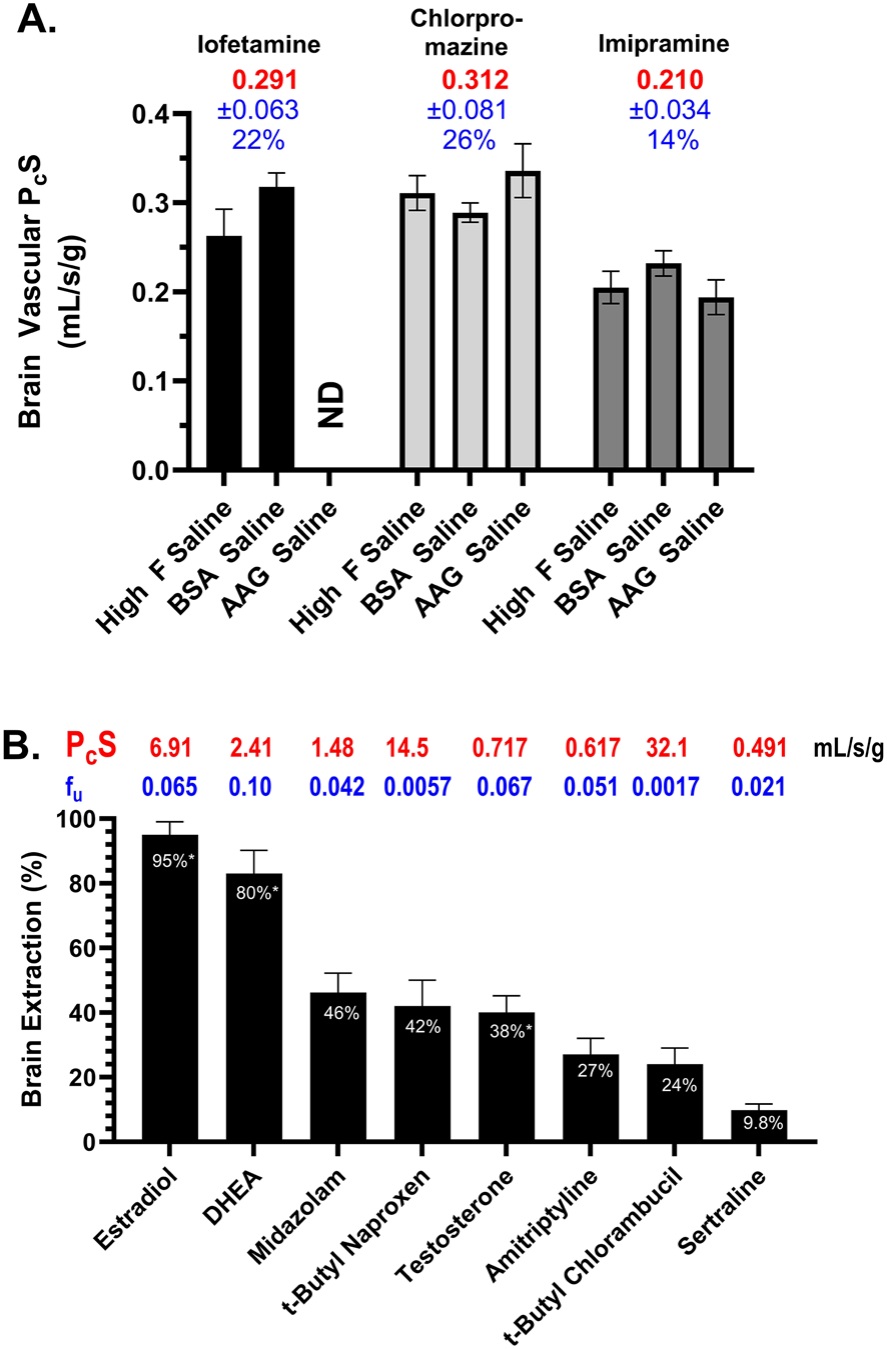
**A** Comparison of P_c_S values measured with the elevated flow method as well as with elevated flow and plasma protein using albumin and alpha acid glycoprotein. Differences were not statistically significant (*P* < 0.05). **B** Brain extraction, P_c_S values, and f_u_ are shown for a series of the compounds that exhibited the highest brain vascular extraction with the elevated flow method.

The two approaches—elevated flow and added plasma protein, were compared (Fig. 12A) with the additional question contrasting serum albumin with alpha-1 acid glycoprotein (AAG) for three solutes—iofetamine, chlorpromazine and imipramine, that fell in ranges accessible to both techniques. Values did not differ significantly across methods for each of the three compounds (*P* > 0.05). Figure 12B illustrates P_c_S values for the highest uptake solutes studied using elevated flow. The combined flow and protein method allowed determination of P_c_S for the highest uptake compounds, like estradiol (6.91 mL/s/g), amitriptyline (0.62), sertraline (0.49), palmitate (2.8) and arachidonate (6.9). Final values of P_o_S and P_c_S are summarized in Tables 2 and 3 for all the compounds studied, assuming a S of 100 cm^2^/g or per cm^3^ measurement (average of five separate determinations).

**Table 2:** Chemical characteristics of compounds

Solute	Log P_oct_	Log D_oct pH=7.4_	MW	f_n_ (%)	P_c_S (mL/s/g)	SD or SE	CV (%)	N	Analysis	Method
Erythrosine B	6.96	−0.15	834	7.8E−06	6.5E−04	6.6E−05	10	9	14C	S 1 min
Arachidonate	7.57	3.79	304	0.22	6.9	1.49	21	12	14C	S 15–60 s
Palmitate	6.96	3.10	256	0.22	2.8	0.34	12	32	14C	S 15–60 s
Chlorpromazine	5.40	3.45	319	1.42	0.31	5.1E−02	17	53	14C	S 15–60 s
t-butyl chlorambucil	5.24	5.24	376	100	32.1	6.84	21	12	HPLC	S 15–60 s
Sertraline	5.00	3.19	306	1.56	0.49	9.5E−02	19	12	3H	S 15–60 s
t-Butyl-naproxen	5.00	5.00	302	100	14.5	2.55	18	12	HPLC	S 15–60 s
amitriptyline	4.61	2.80	277	0.81	0.62	0.11	18	12	3H	S 15–60 s
Fluoxetine	4.50	2.28	309	0.27	0.20	0.06	28	18	3H	S 15–60 s
Imipramine	4.39	2.17	280	0.99	0.21	3.4E−02	16	26	3H	S 15–60 s
Ibuprofen	4.13	1.44	206	0.11	0.034	3.5E−03	10	24	14C	S 15–60 s
Estradiol	3.94	3.94	272	100	6.91	0.76	17	18	14C	S 15–60 s
Flurbiprofen	3.99	0.91	224	0.06	2.3E−02	3.5E−03	15	25	14C	S 15–60 s
Warfarin	3.54	1.12	308	0.01	7.1E−03	7.5E−04	11	32	14C	S 15–60 s
Indomethacin	3.51	0.68	358	0.05	7.7E−03	1.6E−03	21	41	14C	S 15–60 s
Paroxetine	3.50	1.00	329	0.32	0.12	2.4E−02	19	28	14C	S 15–60 s
Progesterone	3.48	3.48	315	100	3.5	4.0E−01	11	22	3H	S 15–60 s
Propranolol	3.48	1.41	259	0.74	0.10	3.2E−02	33	34	14C	S 15–60 s
Chlorambucil	3.41	0.61	304	0.16	2.9E−02	4.6E−03	16	28	14C	S 15–60 s
Naproxen	3.24	0.09	230	0.05	6.9E−03	9.4E−04	14	34	14C	S 15–60 s
Iofetamine	3.26	1.75	299	3.07	0.29	6.3E−02	22	22	14C	S 15–60 s
Diphenhydramine	3.18	1.39	255	1.96	8.5E−02	1.9E−02	23	18	3H	S 15–60 s
Midazolam	3.12	3.12	326	99.0	1.5	3.8E−01	26	20	14C	S 15–60 s
Testosterone	3.1	3.1	288	100	0.74	1.0E−01	14	26	14C	S 15–60 s
DHEA	3.00	3.00	288	100	3.2	5.2E−01	16	24	14C	S 15–60 s
Diazepam	2.84	2.84	285	100	0.95	5.9E−02	7	189	14C & 3H	S 10 s–25 min
Triasolam	2.71	2.71	342	100	0.73	1.4E−01	20	18	3H	S 10–20 s
Alprasolam	2.63	2.63	309	100	0.49	1.7E−01	35	24	3H	S 10–20 s
Valproic acid	2.71	0.21	144	0.16	9.2E−03	1.7E−03	18	24	14C	S 10–20 s
Lorazepam	2.39	2.39	321	100	0.30	1.3E−01	32	18	3H	S 10–20 s
Kynurenic acid	2.28	−2.06	189	4.6E−7	1.2E−04	2.2E−05	18	10	3H	S 30 s
Oxazepam	2.37	2.37	287	100	0.23	6.6E−02	28	20	3H	S 10–20 s
Tolbutamide	2.34	1.00	270	0.61	3.5E−03	5.5E−04	16	24	14C	S 15–60 s
N-butyl-benzene sulfonamide	2.20	2.20	213	100	0.38	9.8E−02	26	36	LCMS	S 5–60 s
Pentobarbital	2.08	2.01	226	75.1	4.5E−02	1.8E−03	4	16	14C	S 15–60 s
Phenytoin	2.24	2.17	252	88.4	2.6E−02	2.0E−03	7	34	14C	S 15–60 s
Flunitrazepam	2.03	2.03	313	100	0.12	2.5E−02	22	45	14C	S 15–60 s
Temazepam	2.19	2.19	301	100	0.13	3.1E−02	24	18	3H	S 15–60 s
Reserpine	1.54	1.46	609	83.37	9.0E−02	9.3E−03	10	12	3H	S 10–20 s
Cyclosporin	1.86	1.86	1023	100	2.4E−02	3.4E−03	14	18	14 C	S 5–30 s
Hexanol	2.03	2.03	102	100	0.18	2.4E−02	14	12	3H	S 5–20 s
Phenobarbital	1.53	1.51	232	47.7	6.5E−03	1.1E−03	16	24	14C	S 5–15 s
Nicotine	1.32	0.45	162	44.3	0.12	2.6E−02	22	24	14C	S 15–60 s
Iodoantipyrine	1.27	1.27	314	100	0.16	2.8E−02	18	38	14C	S 15–60 s
Anthranilic acid	1.21	−1.27	137	0.33	1.2E−03	2.6E−04	22	10	14C	S 30 s
Aminopyrine	1.00	1.00	231	99.0	5.47E−02	9.8E−03	18	18	14C	S 15–60 s
3-hydroxy-anthranilic acid	1.05	−1.78	153	0.15	1.3E−04	1.4E−05	11	10	14C	S 30 s
Butanol	0.88	0.88	74	100	0.15	4.2E−02	29	28	14C	S 10–30 s
Propanol	0.25	0.25	60	100	7.9E−02	1.7E−02	22	15	14C	S 10 s
1,6-Hexanediol	0.83	0.83	118	100	3.8E−02	6.6E−03	17	16	14C	S 20 s
Antipyrine	0.38	0.38	188	100	2.4E−02	5.4E−03	23	86	14C	S 15–120 s
Theophylline	0.00	0.00	180	100	2.8E−03	4.9E−04	18	12	14C	S 30 s
Barbital	0.65	0.64	184	79.9	3.8E−03	3.6E−04	9	8	14C	S 10 s
Caffeine	−0.01	−0.01	194	100	2.5E−02	3.6E−03	14	10	14C	S 15 s
Ethanol	−0.23	−0.23	46	100	4.3E−02	5.3E−03	13	18	14C	S 30 s
Iodoacetamide	−0.19	−0.19	185	100	9.2E−03	8.7E−04	9	47	14C	S 7, 14, 21, 35 s
Nicotinamide	−0.34	−0.34	122	100	2.5E−03	3.8E−04	15	12	14C	S 30 s
Methanol	−0.52	−0.52	32	100	2.8E−02	5.2E−03	19	12	14C	AB 15–30 s
Water	−0.59	−0.59	18	100	2.9E−02	2.1E−03	7	36	3H	AB 15–30 s
1,4-Butanediol	−0.52	−0.52	90	100	1.5E−02	1.4E−03	9	9	14C	S 40 s
1,2-Propanediol	−1.04	−1.04	76	100	2.2E−03	2.6E−04	12	36	14C	S 40 s
1,3 Propanediol	−0.92	−0.92	76	100	5.2E−03	1.3E−03	25	12	14C	S 40 s
Temozolamide	−1.04	−1.04	194	100	1.6E−03	3.7E−04	23	24	3H	
Thiourea	−1.02	−1.02	60	100	4.0E−04	2.5E−05	6	47	14C	S 20–60 s
Acetamide	−1.26	−1.26	59	100	9.6E−04	1.3E−04	13	10	14C	S 40 s
Ethylene glycol	−1.36	−1.36	62	100	5.7E−04	1.1E−04	19	37	14C	S 20–60 s
Methylurea	−1.40	−1.40	74	100	1.9E−04	3.3E−05	17	4	14C	S 60 s
Quinolinic acid	−1.44	−4.08	167		1.7E−06	6.4E−06	376	6	3H	S 1–5 min
Glycerol	−1.76	−1.76	92	100	8.3E−05	6.3E−06	8	12	14C	AB 120 s
Urea	−1.66	−1.66	60	100	9.3E−05	1.6E−05	17	36	14C	S 30–120 s
Erythritol	−2.29	−2.29	122	100	3.2E−05	8.0E−06	22	8	14C	S 15, 60 s
l-Glucose	−3.18	−3.18	180	100	1.25E−05	3.1E−07	15	12	14C	S 15, 60 s
Mannitol	−3.10	−3.10	182	100	8.8E−06	6.3E−07	11	18	14C	S+AB 1–5 min
Fructose	−3.20	−3.20	180	100	5.74E−06	6.2E−07	12	6	14C	S+AB 1–5 min
Sucrose	−3.57	−3.57	342	100	4.81E−06	1.1E−06	44	24	14C	S+AB 1–10 min
N-acetyl-cysteine	−3.70	−3.70	163		1.82E−06	4.9E−07	27	14	14C	S+AB 1-5 min
Raffinose	−3.89	−3.89	542	100	9.5E−07	3.4E−07	59	9	3H	S+AB 1-15 min
Inulin	−4.36	−4.13	5000	89.0	7.5E−07	7.8E−08	33	18	C14	S+AB 1-15 min

P_oct_ and D_oct_ values obtained from Hansch et al. [123] Avdeef [46] or from other sources; S = Physiological Saline, AB = Artificial Blood

Flow used for PS was within the range of 0.1–0.25 mL/s/g. S was assumed to be 100 cm^2^/(g (or cm^3^))

**Table 3:** Permeability characteristics of compounds

Solute	P_o_S (mL/s/g)	Log P_o_S	P_c_ (cm/s)	P_o_(cm/s)	Log P_c_	Log P_o_
Erythrosine B	8348	3.92	6.53E−06	83.48	−5.19	1.92
Arachidonate	3107	3.49	6.94E−02	31.07	−1.16	1.49
Palmitate	1254	3.10	2.80E−02	12.54	−1.55	1.10
Chlorpromazine	19.8	1.30	3.09E−03	0.20	−2.51	−0.70
t-butyl chlorambucil	32.1	1.51	3.21E−01	0.32	−0.49	−0.49
Sertraline	31.5	1.50	4.91E−03	0.31	−2.31	−0.50
t-butyl-naproxen	14.5	1.16	1.45E−01	0.15	−0.84	−0.84
Amitriptyline	76.5	1.88	6.17E−03	0.77	−2.21	−0.12
Fluoxetine	74.7	1.87	2.05E−03	0.75	−2.69	−0.13
Imipramine	21.2	1.33	2.10E−03	0.21	−2.68	−0.67
Ibuprofen	30.6	1.49	3.43E−04	0.31	−3.46	−0.51
Estradiol	6.91	0.84	6.91E−02	6.91E-02	−1.16	−1.16
Flurbiprofen	38.2	1.58	2.30E−04	0.38	−3.64	−0.42
Warfarin	51.2	1.71	7.06E−05	0.51	−4.15	−0.29
Indomethacin	14.6	1.16	7.66E−05	0.15	−4.12	−0.84
Paroxetine	39.0	1.59	1.23E−03	0.39	−2.91	−0.41
Progesterone	3.5	0.55	3.54E−02	3.54E−02	−1.45	−1.45
Propranolol	13.21	1.12	9.72E−04	0.13	−3.01	−0.88
Chlorambucil	18.14	1.26	2.87E−04	0.18	−3.54	−0.74
Naproxen	13.07	1.12	6.85E−05	0.13	−4.16	−0.88
Iofetamine	9.49	0.98	2.91E−03	9.49E−02	−2.54	−1.02
Diphenhydramine	2.91	0.46	8.54E−04	2.91E−02	−3.07	−1.54
Midasolam	1.47	0.17	1.46E−02	1.47E−02	−1.84	−1.83
Testosterone	0.74	−0.13	7.40E−03	7.40E−03	−2.13	−2.13
DHEA	3.18	0.50	3.18E−02	3.18E−02	−1.50	−1.50
Diazepam	0.90	−0.05	9.0E−03	9.0E−03	−2.05	−2.05
Triasolam	0.73	−0.14	7.32E−03	7.32E−03	−2.14	−2.14
Alprasolam	0.49	−0.31	4.85E−03	4.85E−03	−2.31	−2.31
Valproic acid	2.92	0.46	9.19E−05	2.92E−02	−4.04	−1.54
Lorazepam	0.30	−0.52	3.0E−03	3.0E−03	−2.52	−2.52
Kynurenic acid	2.60	0.42	1.19E−06	2.60E−02	−5.92	−1.58
Oxazepam	0.23	−0.63	2.33E−03	2.33E−03	−2.63	−2.63
Tolbutamide	0.58	−0.24	3.54E−05	5.78E−03	−4.45	−2.24
N-butyl-benzene sulfonamide	0.38	−0.42	3.80E−03	3.80E−03	−2.42	−2.42
Pentobarbital	5.99E−02	−1.22	4.50E−04	5.99E−04	−3.35	−3.22
Phenytoin	2.98E−02	−1.53	2.63E−04	2.98E−04	−3.58	−3.53
Flunitrazepam	0.11	−0.94	1.14E−03	1.14E−03	−2.94	−2.94
Temazepam	0.13	−0.88	1.31E−03	1.31E−03	−2.88	−2.88
Reserpine	0.11	−0.97	8.98E−04	1.08E−03	−3.05	−2.97
Cyclosporin	2.41E−02	−1.62	2.41E−04	2.41E−04	−3.62	−3.62
Hexanol	0.18	−0.75	1.77E−03	1.77E−03	−2.75	−2.75
Phenobarbital	1.36E−02	−1.86	6.51E−05	1.36E−04	−4.19	−3.86
Nicotine	0.27	−0.57	1.19E−03	2.69E−03	−2.92	−2.57
Iodoantipyrine	0.16	−0.81	1.56E−03	1.56E−03	−2.81	−2.81
Anthranilic acid	0.36	−0.44	1.19E−05	3.60E−03	−4.92	−2.44
Aminopyrine	5.52E−02	−1.26	5.47E−04	5.52E−04	−3.26	−3.26
3-hydroxy-anthranilic acid	8.86E−02	−1.05	1.31E−06	8.86E−04	−5.88	−3.05
Butanol	0.13	−0.90	1.47E−03	1.27E−03	−2.83	−2.90
Propanol	7.90E−02	−1.10	7.90E−04	7.90E−04	−3.10	−3.10
1,6-Hexanediol	3.79E−02	−1.42	3.79E−04	3.79E−04	−3.42	−3.42
Antipyrine	2.37E−02	−1.63	2.37E−04	2.37E−04	−3.63	−3.63
Theophylline	2.78E−03	−2.56	2.78E−05	2.78E−05	−4.56	−4.56
Barbital	4.77E−03	−2.32	3.81E−05	4.77E−05	−4.42	−4.32
Caffeine	2.50E−02	−1.60	2.50E−04	2.50E−04	−3.60	−3.60
Ethanol	4.25E−02	−1.37	4.25E−04	4.25E−04	−3.37	−3.37
Iodoacetamide	9.17E−03	−2.04	9.17E−05	9.17E−05	−4.04	−4.04
Nicotinamide	2.46E−03	−2.61	2.46E−05	2.46E−05	−4.61	−4.61
Methanol	2.78E−02	−1.56	2.78E−04	2.78E−04	−3.56	−3.56
Water	2.87E−02	−1.54	2.87E−04	2.87E−04	−3.54	−3.54
1,4-Butanediol	1.51E−02	−1.82	1.51E−04	1.51E−04	−3.82	−3.82
1,2-Propanediol	2.16E−03	−2.67	2.16E−05	2.16E−05	−4.67	−4.67
1,3 Propanediol	5.16E−03	−2.29	5.16E−05	5.16E−05	−4.29	−4.29
Temozolamide	1.63E−03	−2.79	1.63E−05	1.63E−05	−4.79	−4.79
Thiourea	3.98E−04	-3.40	3.98E−06	3.98E−06	−5.40	−5.40
Acetamide	9.63E−04	−3.02	9.63E−06	9.63E−06	−5.02	−5.02
Ethylene glycol	5.69E−04	−3.24	5.69E−06	5.69E−06	−5.24	−5.24
Methylurea	1.91E−04	−3.72	1.91E−06	1.91E−06	−5.72	−5.72
Quinolinic acid	7.43E−04	−3.13	1.70E−08	7.43E−06	−7.77	−5.13
Glycerol	8.29E−05	−4.08	8.29E−07	8.29E−07	−6.08	−6.08
Urea	9.28E−05	−4.03	9.28E−07	9.28E−07	−6.03	−6.03
Erythritol	3.24E−05	−4.49	3.24E−07	3.24E−07	−6.49	−6.49
l-Glucose	1.25E−05	−4.90	1.25E−07	1.25E−07	−6.90	−6.90
Mannitol	8.80E−06	−5.06	8.80E−08	8.80E−08	−7.06	−7.06
Fructose	5.74E−06	−5.24	5.74E−08	5.74E−08	−7.24	−7.24
Sucrose	4.81E−06	−5.32	4.81E−08	4.81E−08	−7.32	−7.3
N-acetyl-cysteine	1.82E−06	−5.74	1.82E−08	1.82E−06	−7.74	−7.74
Raffinose	9.50E−07	−6.02	9.50E−09	9.50E−09	−8.02	−8.02
Inulin	7.50E−07	−6.12	7.50E−09	7.50E−09	−8.12	−8.12

Flow used for PS was within the range from 0.1 to 0.25 mL/s/g. S was assumed to be 100 cm^2^/(g (or cm^3^)). P_c_S values represent the average of the different studies

Multiple linear regression analysis of 78 compounds with little or no evidence of active efflux transport revealed that both Log P_oct_ and Log MW had statistically significant correlations that aided the quality of the fit. Parameter values were a = 0.910 ± 0.023 for Log P_oct_ and −b = 0.957 ± 0.179 for Log MW with a total correlation coefficient of R^2^ = 0.958 with 75 degrees of freedom (df).

**Fig. 13:**
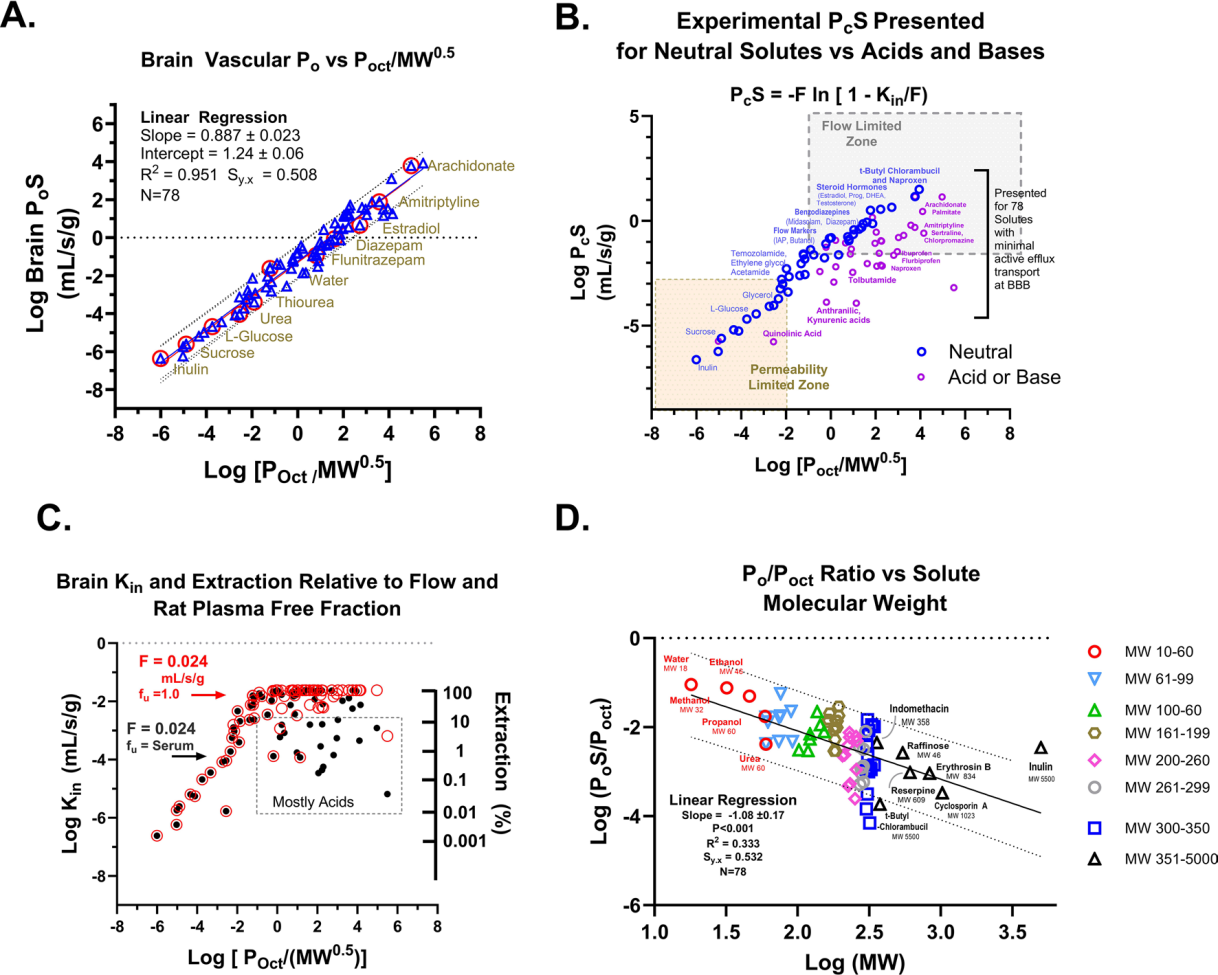
**A** Plot of Log brain vascular intrinsic P_o_S vs. Log [Octanol Partition coefficient/Square root of Molecular Weight] following the Levin and Anderson formalism [104] for 78 drugs, permeability and flow markers, hormones and nutrients. The *line* is the regression best fit to the data. Compounds were all screened to document minimal or no transport contribution by p-glycoprotein and breast cancer resistance protein. Intrinsic P_o_S refers to the permeability of the neutral (unionized) species of the compound. The octanol/water partition coefficient adjusts for the ability of the test molecule to enter or partition in the lipid environment of the cell membrane, The inverse square root of molecule weight (MW) adjusts for the rate of diffusion of the test molecule within the lipid membrane, given as D = constant/MW^0.5^ where D is the diffusion coefficient in bulk. Subsequent work has demonstrated that the square root of MW underrepresents the correction and that a figure closer to MW may be more appropriate. **B** This plot transitions from the intrinsic permeability (P_o_) of the neutral species in **A** to the transcellular permeability for the whole molecule, adjusting for the fraction of solute that is unionized at normal blood pH (7.4). Thus, the plot presents Log P_c_S vs. Log P_oct_/MW^0.5^ where the x-axis is maintained identical to that in **A**. The essential difference in this plot is the marked decrement in PS for acids and bases based upon the fraction that is neutral at physiologic blood pH. Many of the acids and bases (shown in RED) decrease 1–3 orders in magnitude from values for only the neutral species (**A**). For neutral compounds, Log P_o_S = LogP_c_S because f_n_ for neutral solutes equals 1.00. **B**, therefore represents the more appropriate view for PS for whole compound at physiologic pH. **C** In this plot, data from **B** are further processed to include impact of brain flow rate (F) and of plasma protein binding (f_u_). The y-axis on the left side represents Log K_in_ where K_in_ = F × (1 − exp(−f_u_ × P_c_S/F). On the right y-axis, values are given in terms of Log Extraction (E = K_in_/F). The figure illustrates the limit placed on highly lipophilic compounds by blood flow, where K_in_ maximizes to flow rate (F). Similarly, it also shows the marked impact of plasma protein binding on brain uptake for solutes where f_u_ × P_c_S is less than F such that they show “restrictive” plasma protein binding where E < f_u_. The x-axis is identical to **A** and **B**: Log P_oct_/MW^0.5^. *Red outlined circles* represent K_in_ at normal rat flow (0.024 mL/s/g) with no assumed plasma protein binding (f_u_ = 1). *Black circles* represent K_in_ where both flow and f_u_ are at normal in vivo rat values. **D** The plot is changed substantially to focus on the contribution of size or molecular weight to the permeability coefficient at the in vivo brain vascular endothelial cell membrane. On the y-axis, intrinsic P_o_S is normalized to P_oct_ to obtain Log (P_o_S/P_oct_), which are plotted versus Log MW. Compounds in the plot are segregated by MW into groups which are given different symbols. The plot emphasizes the marked impact of size as reflected in each of the groups as values increase in Log MW. The quality of the fit is given in the figure with an R^2^ that is highly significant.

Figure 13A illustrates the best fit of Log BBB P_o_S vs. Log (P_oct_/MW^0.5^) following the Collander formalism with R^2^ = 0.954). Conversion from Log P_o_S to Log P_c_S by multiplication of P_o_S by f_n_ provided the plot in Fig. 13B and shows the marked impact of ionization on the direct correlation where, depending upon pK_a_, compounds differ between P_c_S and P_o_S by twofold to 1000-fold (Fig. 13B). Figure 13C plots calculated K_in_ and E utilizing normal values of rat F and rat f_u_ for calculation. The influence of molecular weight was brought out by normalizing P_o_S values by dividing by Log P_oct_ and then correlating the normalized P_o_S value to Log molecular weight (MW) (Fig. 13D). Figure 13C illustrates K_in_ or E values at F = 0.024 mL/s/g with some values deviating downward based upon protein binding or percent ionized. The Log (P_o_S/P_oct_) relation vs. Log MW in Fig. 13D was statistically significant with R^2^ = 0.333 (*P* < 0.001).

**Fig. 14:**
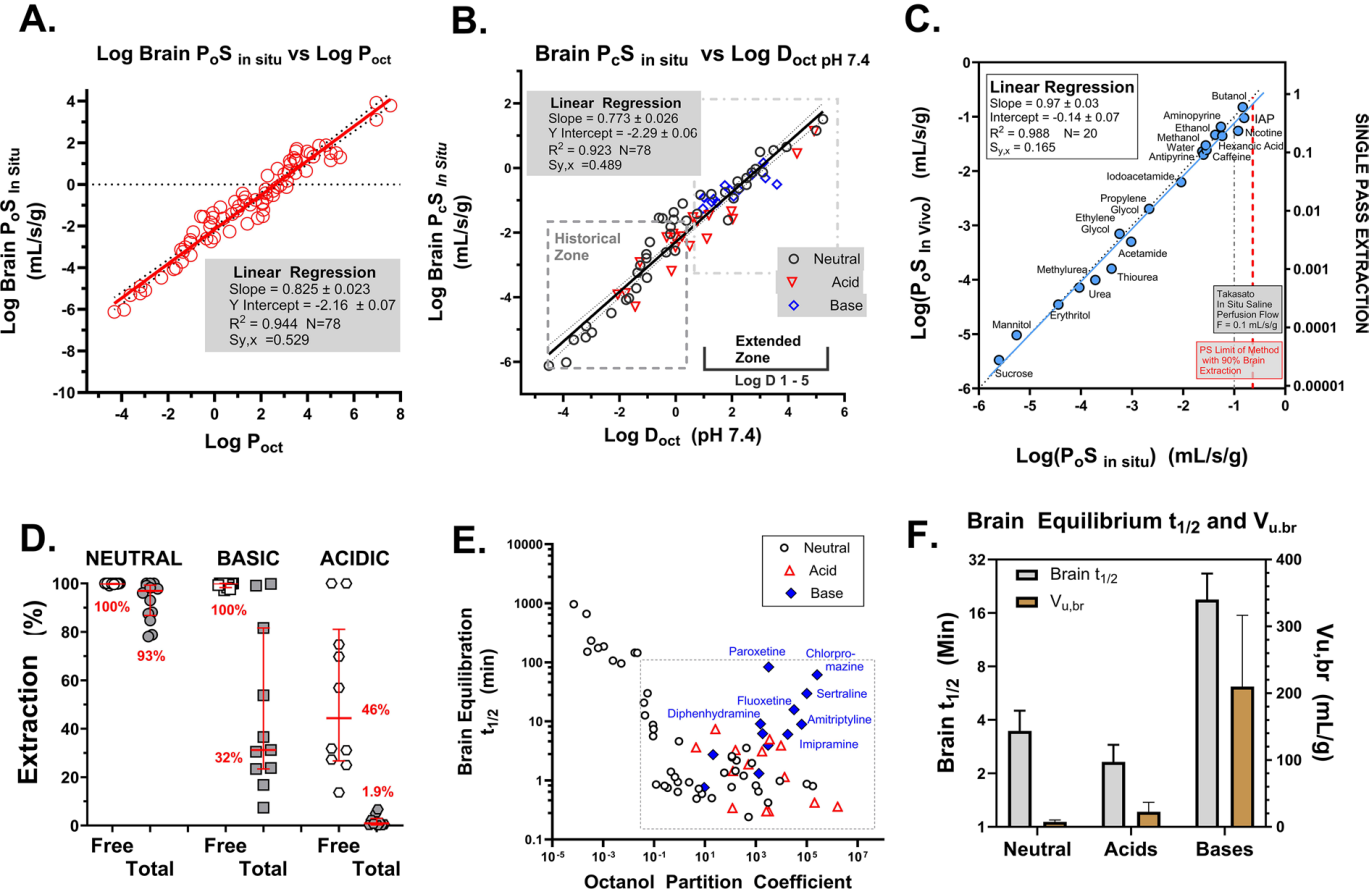
**A** Plot of Log brain vascular intrinsic P_o_S vs. Log Octanol Partition coefficient for 78 drugs, permeability and flow markers, hormones and nutrients. Line is the regression best fit to data. **B** Plot of Log P_c_S (reflecting uptake by both neutral and ionized species) vs. Log Distribution coefficient for octanol at pH 7.4 (D_oct 7.4_). **C** Plot of in vivo BBB P_o_S from database compiled by Fenstermacher and Rapoport [91] plotted against Log P_o_S in situ reported in the present paper. *Solid line* shows the linear regression best fit. **D** Free and total E for neutral, basic, and acid compounds. **E** Brain equilibration t_1/2_ vs. Octanol partition coefficient for compounds identified as neutral, acid or base. Most bases are also labeled. The *dashed horizontal line* is 100 min. **F** Bar plot of brain t_1/2_ and V_u,br_ for neutral, acidic, and basic compounds

Figure 14A, B show matching linear relations between Log P_o_S (R^2^ = 0.944) and Log P_oct_, Log P_c_S and Log D_oct pH7.4_ (R^2^ = 0.923) over 10 orders of magnitude. A strong positive correlation (R^2^ = 0.988) (Fig. 14C) was found over the in vivo range for Log BBB P_o_S in vivo with Log BBB P_o_S in situ using literature values for P_o_S in vivo up to Log P_oct_ ~ 0 compiled by Fenstermacher and Rapoport [91, 92] (Fig. 14C).

Figure 14D exhibits the marked brain E differences that were observed between neutral drugs, lipophilic bases and lipophilic acids. Brain free drug extraction (E_free_), illustrating the effect of the compound itself, is ~100% among the lipophilic neutral drugs and bases tested, whereas acids are ~50% lower. The difference becomes even greater when total extraction (E_total_), is measured in the presence of plasma protein. E_total_ held at near 100% for neutral drugs in the lipophilic range (93% mean), was 32% for basic drugs (~threefold reduction mean E_total_ relative to E_free_) and 37 times lower to only 1.3% E_total_ for acidic drugs. Serum albumin binds acids with particular affinity [13] relative to bases and neutral drugs, which contributes to the lower E_total_ through the Crone Renkin equation.

Acids and neutral drugs also exhibited significantly faster mean calculated brain t_½_ (<5 min) for neutrals and acids vs. >15 min for bases (Fig. 14E, F), related to the larger brain distribution volume and lower plasma protein binding for bases relative to acids.

Table 4 presents calculated K_in_, E, f_u_, and V_u,br_ at a normal physiologic brain flow rate (1.5 mL/min/g) and serum f_u_ for the rat. Literature values from Avdeef and other sources were used for brain f_u_ and V_br_ [46]. The model is based upon drug binding in serum. Some hormones and basic drugs show additional binding to serum globulins, which is not addressed in this manuscript. It is being addressed in a follow up manuscript under preparation for publication.

**Table 4:** Parameters leading to t_1/2_ of compounds

Solute	P_o_S (mL/s/g)	f_u,.p_	Brain blood flow (mL/s/g)	Brain K_in_ (mL/s/g)	Extraction (unbound) (%)	Brain vascular extraction (total) (%)	Brain V_u,br_ (mL/g)	Brain t_1/2_ (min)
Erythrosine B	8348	0.01	0.024	6.53E−06	2.7	0.027	1	17.7
Arachidonate	3107	6.9E−05	0.024	1.21E−03	100	2.0	100	0.17
Palmitate	1254	8.5E−05	0.024	4.44E−04	100	1.0	50	0.21
Chlorpromazine	19.8	0.0354	0.024	8.85E−03	100	36.6	1316	61.1
t-Butyl chlorambucil	32.1	2.0E−03	0.024	2.23E−02	100	93.1	100	0.10
Sertraline	31.5	0.013	0.024	5.60E−03	100	23.4	1108	29.6
t-Butyl-naproxen	14.5	7.5E−03	0.024	2.37E−02	100	98.9	50	0.18
Amitriptyline	76.5	0.03	0.024	1.29E−02	100	53.8	333.3	8.94
Fluoxetine	74.7	0.028	0.024	1.05E−02	100	21.3	250	15.8
Imipramine	21.2	0.021	0.024	2.97E−03	100	16.8	100	6.01
Ibuprofen	30.6	0.016	0.024	5.42E−04	76.0	2.3	3.38	1.15
Estradiol	6.91	0.02	0.024	2.34E−02	100	97.6	100	0.98
Flurbiprofen	38.2	6.3E−03	0.024	1.44E−04	61.6	0.60	7.76	3.90
Warfarin	51.2	8.0E−03	0.024	5.64E−05	25.5	0.24	3.03	4.96
Indomethacin	14.6	5.7E−03	0.024	4.37E−05	27.3	0.18	13.0	19.6
Paroxetine	39.0	0.015	0.024	1.64E−03	99.4	7.4	852.0	83.0
Progesterone	3.5	0.03	0.024	2.37E−02	100	98.8	28.6	0.42
Propranolol	13.21	0.09	0.024	8.06E−03	98.3	30.5	27.8	3.93
Chlorambucil	18.14	0.01	0.024	2.85E−04	69.8	1.2	3.03	1.23
Naproxen	13.07	0.005	0.024	3.42E−05	24.8	0.14	1.85	3.11
Iofetamine	9.49	0.25	0.024	2.40E−02	100	95.2	25	3.16
Diphenhydramine	2.91	0.48	0.024	1.97E−02	97.2	81.9	32.3	9.09
Midasolam	1.47	0.074	0.024	2.11E−02	100	98.9	40.0	1.44
Testosterone	0.74	0.022	0.024	1.18E−02	100	49.3	28.6	0.61
DHEA	3.18	0.05	0.024	2.40E−02	100	99.9	28.65	0.69
Diazepam	0.90	0.133	0.024	2.39E−02	100	99.2	21.3	1.37
Triasolam	0.73	0.239	0.024	2.40E−02	100	99.9	16.7	1.92
Alprasolam	0.49	0.349	0.024	2.40E−02	100	99.9	14.3	2.40
Valproic acid	2.92	0.18	0.024	1.60E−03	31.8	6.7	1.43	1.86
Lorazepam	0..30	0.16	0.024	2.06E−02	100	86	15.87	1.39
Kynurenic acid	2.60	1.00	0.024	1.19E−04	0.49	0.49		
Oxazepam	0.23	0.17	0.024	1.89E−02	100	80.9	20	2.03
Tolbutamide	0.58	0.049	0.024	1.73E−04	13.7	0.72	1.0	3.27
N-butyl-benzene sulfonamide	0.38	0.30	0.024	2.38E−02	100	99.1	10	1.45
Pentobarbital	5.99E−02	0.60	0.024	1.62E−02	84.7	67.5	3.38	1.44
Phenytoin	2.98E−02	0.15	0.024	3.64E−03	66.6	15.2	12.7	6.02
Flunitrazepam	0.11	0.19	0.024	1.88E−02	99.1	60.0	12.5	1.93
Temazepam	0.13	0.18	0.024	1.50E−02	99.6	62.4	18.5	2.57
Reserpine	0.11	0.10	0.024	7.49E−03	97.6	31.2	5	0.77
Cyclosporin	2.41E−02	0.04	0.024	8.29E−04	63.4	3.5		
Hexanol	0.18	0.30	0.024	2.14E−02	99.9	89.1	8.33	1.35
Phenobarbital	1.36E−02	0.25	0.024	1.57E−03	23.8	6.6	4.03	7.39
Nicotine	0.27	0.87	0.024	1.71E−02	99.3	98.7	5.88	2.51
Iodoantipyrine	0.16	0.50	0.024	2.31E−02	99.8	96.1	2	0.50
Anthranilic acid	0.36	0.50	0.024	5.89E−04	4.8	2.5		
Aminopyrine	5.52E−02	0.80	0.024	2.01E−02	89.8	83.9	1.67	0.76
3-hydroxy-anthranilic acid	8.86E−02	1.00	0.024	1.31E−04	0.54	0.54		
Butanol	0.13	0.60	0.024	2.29E−02	99.8	97.5	2	0.59
Propanol	7.90E−02	0.74	0.024	2.19E−02	96.3	91.2	1.27	0.25
1,6-Hexanediol	3.79E−02	0.9	0.024	1.82E−02	96.3	75.9	1.27	0.72
Antipyrine	2.37E−02	0.92	0.024	1.43E−02	62.7	59.7	1.25	0.93
Theophylline	2.78E−03	0.9	0.024	2.38E−03	10.9	9.9	1.05	4.60
Barbital	4.77E−03	0.985	0.024	3.47E−03	14.7	14.5	1.11	3.63
Caffeine	2.50E−02	0.96	0.024	1.52E−02	64.7	63.2	1.28	0.94
Ethanol	4.25E−02	0.95	0.024	1.95E−02	83.0	81.4	1.14	0.64
Iodoacetamide	9.17E−03	0.92	0.024	1.82E−06	31.7	29.6	1.11	1.66
Nicotinamide	2.46E−03	1	0.024	7.11E−03	9.7	9.7	1.04	5.14
Methanol	2.78E−02	1.00	0.024	2.34E−03	68.6	68.6	1.06	0.75
Water	2.87E−02	1.00	0.024	1.65E−02	69.8	69.8	1.18	0.81
1,4-Butanediol	1.51E−02	1.00	0.024	1.67E−02	46.7	46.7	1.11	1.14
1,2-Propanediol	2.16E−03	1.00	0.024	1.12E−02	8.6	8.6	1.04	5.82
1,3 Propanediol	5.16E−03	1.00	0.024	2.07E−03	19.3	19.3	1.02	2.53
Temozolamide	1.63E−03	0.80	0.024	4.64E−03	4.3	5.3	1.05	7.65
Thiourea	3.98E−04	1.00	0.024	1.27E−03	1.6	1.6	1.03	30.1
Acetamide	9.63E−04	1.00	0.024	3.95E−04	3.9	3.9	1.05	12.9
Ethylene glycol	5.69E−04	1.00	0.024	9.44E−04	2.3	2.3	1.02	20.9
Methylurea	1.91E−04	1.00	0.024	5.63E−04	0.79	0.79	1.04	63.1
Quinolinic acid	7.43E−04	1.00	0.024	1.90E−04	0.01	0.0071		
Glycerol	8.29E−05	1.00	0.024	1.70E−06	0.34	0.34	1.04	145.2
Urea	9.28E−05	1.00	0.024	8.27E−05	0.39	0.39	0.77	95.7
Erythritol	3.24E−05	1.00	0.024	9.27E−05	0.15	0.15	0.50	160.3
l-Glucose	1.25E−06	1.00	0.024	1.25E−05	5.21E-04	5.21E-04	0.13	116
Mannitol	8.80E−06	1.00	0.024	8.80E−06	3.67E-04	3.67E-04	0.08	109
Fructose	5.74E−06	1.00	0.024	5.74E−06	2.39E-04	2.39E-04	0.08	168
Sucrose	4.817E−06	1.00	0.024	4.81E−06	2.00E-04	2.00E-04	0.05	120
N-acetyl-cysteine	1.82E−06	1.00	0.024	1.82E−06	7.60E-05	7.60E-05	0.05	317
Raffinose	9.50E−07	1.00	0.024	9.50E−07	3.96E-05	3.96E-05	0.03	405
Inulin	7.50E−07	1.00	0.024	7.50E−07	3.12E-05	3.12E-05	0.02	308

f_u,p_, f_u,br_, and V_u,br_ are from rat in vivo measurements

**Fig. 15:**
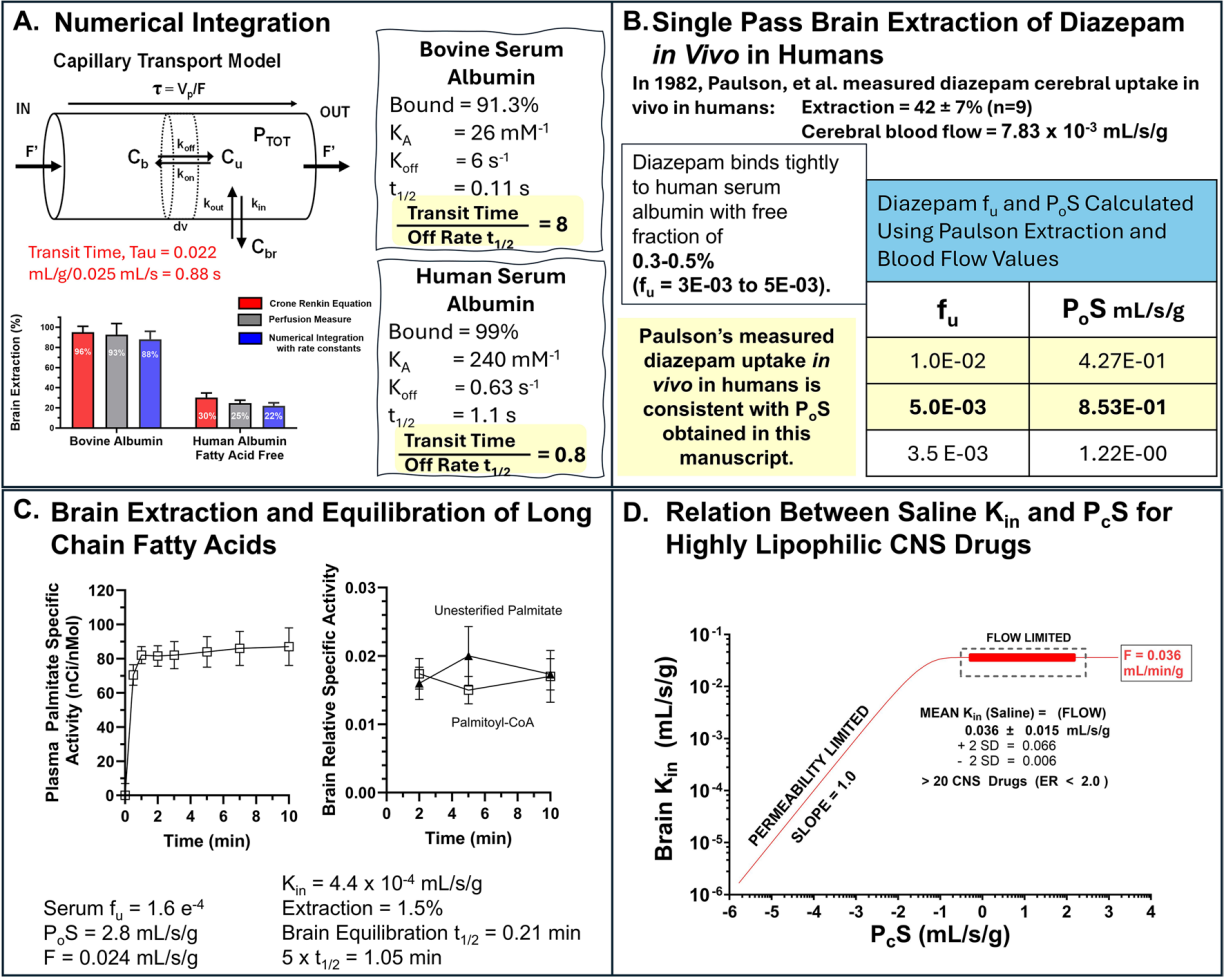
**A** Diagram comparing brain diazepam extraction during perfusion with 2.7% bovine or human serum albumin. Schematic model of a brain capillary with progressive exchange between plasma protein bound and free diazepam pools as well as brain uptake and back flux. Modeling showed that diazepam completed >8 cycles of binding and dissociation with lower affinity bovine albumin during a single pass through the capillary bed and had a brain extraction of >90%. In contrast, higher affinity fatty acid free human albumin was associated with only a single cycle and brain extraction of ~20–30%. **B** Data showing that substitution of this study’s diazepam P_o_S into the PET study of Paulson et al. [41] provides a predicted free fraction in plasma that matches precisely with that measured for humans in vivo (0.5–1%). **C** Illustration of time course of infusion of fatty acid tracer and time course of tracer equilibration in brain fatty acid and palmitoyl-CoA pools (plots adapted from Grange et al. [65]). **D** Crone Renkin relationship between K_in_ vs. PS_in_ for a F of 0.033 mL/s/g. In the figure, information is provided for mean K_in_ and SD (0.036 ± 0.015 mL/s/g) of the top 20 drugs, using data from Summerfield [31]. The *dashed box* delineates the zone where 94% of the values are expected (representing ± 2 SD around the mean K_in_). The plot reinforces the importance of F to the determination of K_in_ for lipophilic drugs from saline.

Figure 15A presents values of brain diazepam extraction measured using bovine and human serum compared to the Crone Renkin values and to that calculated by numerical integration, taking into account on and off rates of binding to the plasma protein [44]. Bovine albumin had a 93% measured extraction, compared with 96% by Crone Renkin and 88% by numerical integration. The measured extraction from bovine serum albumin was quite high (93%); within 10% of that predicted by both the Crone Renkin equation and numerical integration, using a dissociation k_off_ of 6 s^−1^ or greater (predicted range 4–8 s^−1^). The calculated t_1/2_ for dissociation (0.11 s) allowed eight cycles of dissociation and rebinding for each transit (0.3–1 s at normal flow) through the brain capillary network from the arterial to the venous side. In contrast, the measured extraction with fatty-acid free human serum albumin was far less than with bovine serum albumin, less than one complete dissociation cycle per capillary transit. The predicted k_off_ range (0.4–0.8 s^−1^) for human albumin matched that measured in vitro by Zheng et al. (0.63 s^−1^) [53]. The lower diazepam extraction with human albumin was largely determined by the tenfold greater human albumin K_A_ (2.4–2.6 × 10^5^ M^−1^) than bovine albumin (not fatty acid free) (K_A_ > 2.5 × 10^4^ M^−1^). The bovine albumin was not fatty acid free and contained small levels of associated fatty acids that could influence the apparent K_A_. The perfusion fluid f_u_ for diazepam with bovine albumin more closely matches rat serum in vivo f_u_ (0.10–0.15). Figure 15B shows that insertion of our P_o_S value into the Crone Renkin equation, provides an excellent fit of the model to the data, with a free fraction fully in line with the human value (0.5–1.5%). Further, Fig. 15C shows that predicted time for free palmitate and arachidonate equilibration in the brain unesterified and palmitoyl-CoA precursor pools (4–5 × t_1/2_) matches that measured in vivo (i.e., brain t_1/2_ ~ 0.20 min, ×4–5 for equilibration = 1.0–1.2 min [64, 65]). Figure 15D shows the Crone Renkin relationship between brain K_in_ and BBB P_c_S and illustrates the flow-limited plateau that exists in the high permeability and lipophilicity range of CNS drugs, when measured in the absence of plasma protein. The mean K_in_ value of the top 20 compounds from Summerfield et al. [31] is 0.036 mL/s/g, which is virtually identical to that measured by Summerfield with diazepam (0.033 mL/s/g).

Table 5 lists ~25 P_c_S values for a series of solutes studied in our laboratory that show facilitated transport into brain at the BBB by Lat-1, Ent-1, or Oatp. For Lat-1 and Ent-1, values are presented for brain uptake P_s_C at tracer concentrations. In the Oatp case, P_c_S is also shown for a solute (paclitaxel), which is known to exhibit marked active efflux transport by BBB P-glycoprotein. Inhibition of P-glycoprotein transport by elacridar markedly increases BBB paclitaxel influx. However, the same does not occur in Oatp knockout animals, showing the importance of Oatp in brain paclitaxel influx.

**Table 5:** Characteristics of other classes of compounds

LAT1	Solute	Log Poct	Log D_oct_ 7.4	MW	PS (mL/s/g)	SD	LogPS	N	Analysis	Method
l-Phenylalanine	AA	1.110	−1.46	165	7.80E−02	1.68E−02	−1.108	7	14C	S 15 s
l-DOPA	Drug	−0.22	−2.7	197	1.02E−02	1.95E−04	−1.99	6	3H	S 15 s
Acivicin	Neurotoxic Drug	−0.31	−2.63	179	4.79E−04	7.65E−05	−3.32	6	3H	S 30 s
l-BMAA	Neurotoxic Drug	−0.85	−3.4	118	5.42E−04	1.08E−04	−3.27	6	GCMS	
Gabapentin	Amino Acid	1.19	−1.4	171	4.73E−03	9.53E−04	−2.33	8	3H	S 15 s
Pregabalin	Drug	1.12	−1.75	159	9.76E−04	1.33E−04	−3.01	3	3H	S 15 s
Melphalan (para)	Drug	1.79	−1.03	305	1.04E−03		−2.98		14C	20 s 10 s w
Meta-sarcolysine	Drug	1.79	−1.03	305	5.66E−02	2.40E−03	−1.25	10	3H	20 s 10 s w
d,l-6-NAM	Drug (para)	2.36	−0.33		4.42E−02	1.36E−03	−1.35	8	3H	S 30 s
d,l-7-NAM	Drug (meta)	2.36	−0.22		4.42E−02	1.36E−03	−1.35	8	3H	S 30 s

ENT1	Solute	Log Poct	Log D_oct_ 7.4	MW	PS (mL/s/g)	SD	LogPS	N	Analysis	Method
Gemcitabine	Drug	−0.470	−1.36	263	1.64E−05	9.20E−06	−4.785	11	3H	S 30–120 s
5ʹ-deoxy-5-fluorouridine (5ʹ-DFUR)	Metabolite				3.20E−04	3.12E−05	−3.49	6	3H	S 30–120 s
Capecitabine	Drug	0.97	−0.96	359	1.40E−04		−3.85	12	3H	S 30–120 s
5-FU	Drug	−0.78	−1.57	130	5.01E−05	1.50E−05	−4.30	4		S 30–120 s

OATP	Solute	Log Poct	Log D_oct_ 7.4	MW	PS (mL/s/g)	SEM	LogPS	N	Analysis	PS Fold Change
Paclitaxel	Drug	7.38	3.89	854	1.13E−04	4.42E−02	−3.95	15	3H	1
Elacridar (2 μM)	To Raise Signal				6.04E−03	5.10E−04	−2.22	6	3H	53
Elacridar in Oatp KO					4.40E−04	1.20E−04	−3.36	4		3.9

MCT8 l-T3 Transporter	Solute	Log Poct	Log D_oct_ 7.4	MW	PS (mL/s/g)	SEM	LogPS	N
l-T3	Drug/Hormone				1.46E−02	3.21E−04		8

Facilitated Nutrients									
Transporter	Substrate	Log Poct	Log Doct 7.4	PS	SEM	Log PS	N	Analysis	Method
GLUT1	d-Glucose		−1.88	6.94E−03	1.48E−03	−2.16	5	14C	S 16 s
MAT1	l-Lactate		−0.70	1.25E−03	3.50E−04	−2.90	5	14C	S 30 s
LAT1	l-Leucine		0.73	3.54E−02	6.17E−03	−1.45	8	14C	
CAT1	l-Lysine		−1.04	5.21E−03	9.40E−04	−2.28	6	14C	
	l-Glutamate			1.51E−04		−3.82			
Choline transporter	Choline		−3.70	1.30E−03		−2.89			S 20 s
Nucleoside	Adenosine		−1.02	1.70E−03	3.34E−04	−2.77	6		S 15–120 s
Nitrogen base	Adenine		−2.12						

Table 6 shows calculated F values from the Crone Renkin equation at 90% extraction for saline and for plasma for the top 20 compounds of this study—ranked in order from high to low F value. Table 7 shows matched agreement between predicted and measured t_1/2_ for 16 compounds tested in our laboratory or in the literature for rat or matched experimental animal.

**Table 6:** 90% extraction with maximal flow rate in saline and saline + plasma protein for top 20 compounds

Saline			Saline + plasma protein		
Maximal flow—90% extraction			Maximal flow—90% extraction		
Sort	Solute	P_c_S/2.3 mL/s/g	Sort	Solute	f_u,p_ × P_c_S/2.3 mL/s/g
1	t-butyl chlorambucil	14.0	1	Triasolam	0.076
2	t-butyl-naproxen	6.3	2	Alprasolam	0.074
3	Arachidonate	3.0	3	DHEA	0.069
4	Estradiol	2.0	4	Diazepam	0.050
5	Progesterone	1.5	5	N-butyl-benzene sulfonamide	0.050
6	DHEA	1.4	6	t-butyl-naproxen	0.047
7	Palmitate	1.2	7	Midazolam	0.047
8	Midazolam	0.63	8	Progesterone	0.046
9	Diazepam	0.38	9	Nicotine	0.045
10	Testosterone	0.32	10	Estradiol	0.039
11	Triasolam	0.32	11	Butanol	0.038
12	Amitriptyline	0.27	12	Iodoantipyrine	0.034
13	Sertraline	0.21	13	Iofetamine	0.032
14	Alprasolam	0.21	14	t-butyl chlorambucil	0.028
15	N-butyl-benzene sulfonamide	0.17	15	Propanol	0.025
16	Lorazepam	0.13	16	Hexanol	0.023
17	Chlorpromazine	0.13	17	Lorazepam	0.020
18	Iofetamine	0.13	18	Aminopyrine	0.019
19	Oxazepam	0.10	19	Diphenhydramine	0.018
20	Imipramine	0.091	20	Ethanol	0.018

P_c_S measurements are from this lab's studies and f_u,p_ values are from the literature in rat

**Table 7:** Comparison of measured and calculated t_1/2_

Brain Equilibration t_1/2_				
Solute	Measured (min)	Calculated (min)	Ratio	Source
Arachidonate	0.20	0.17	1.2	Washizaki [64]
Palmitate	0.20	0.21	1.0	Grange [65]
Amitriptyline	6.4	8.9	0.7	Ochs [114]
Imipramine	4.1	6,0	0.7	Ochs [114]
Diazepam	2.5	1.4	1.7	Our Lab
Chlorambucil	0.75	1.2	0.6	Our Lab
Naproxen	3.1	3.1	1.0	Our Lab
Pentobarbital	1.15	1.4	0.8	Our Lab
Flunitrazepam	2.3	1.9	1.2	Our Lab, Greenblatt [115]
Water	1	0.81	1.2	Our Lab
Nicotine	2.9	2.5	1.1	Bradbury [116]
Antipyrine	1	0.93	1.1	Our Lab, Ochs [114]
Barbital	3.27	3.6	0.9	Mayer [117]
Ethylene glycol	17	21	0.8	Davson [118]
Thiourea	29	30	1.0	Patlak [76]
Urea	130	96	1.4	Johanson [119]
		Average	1.02	
		SD	0.28	
		SEM	0.07	
		CV	27%	

CV reported from SD in studies ranged from 14 to 28%

Lastly, confusion has arisen in the field related to local cerebral perfusion fluid flow rates and perfusion pressures necessary to maintain physiological conditions*.* In the 1980’s when we developed the heart stopped rat perfusion preparation, we selected the pressure of the Circle of Willis (~80 mm Hg) as the goal for the perfusion pump, which was 10 mL/min using saline fluid. Because of the absence of blood cells and the resulting low viscosity, the saline perfusion fluid had a ~fourfold greater flow rate in brain vessels (4 × 1.5 = 6 mL/min/g or 0.1 mL/s/g), but the pressure was the same and shear stress was comparable, because shear stress is proportional to flow × viscosity [93]. We then duplicated the same setting in the other three preparations with pump infusion rates as follows: (a) rat carotid with PPA open = 20 mL/min; (b) mouse carotid with open PPA—5 mL/min and (c) mouse trans cardiac—20 mL/min. A rat trans cardiac method was established years ago, but the necessary flow rate (80 mL/min) was sufficiently high to merit mastering the simple surgery to do the carotid artery preparation.

**Fig. 16:**
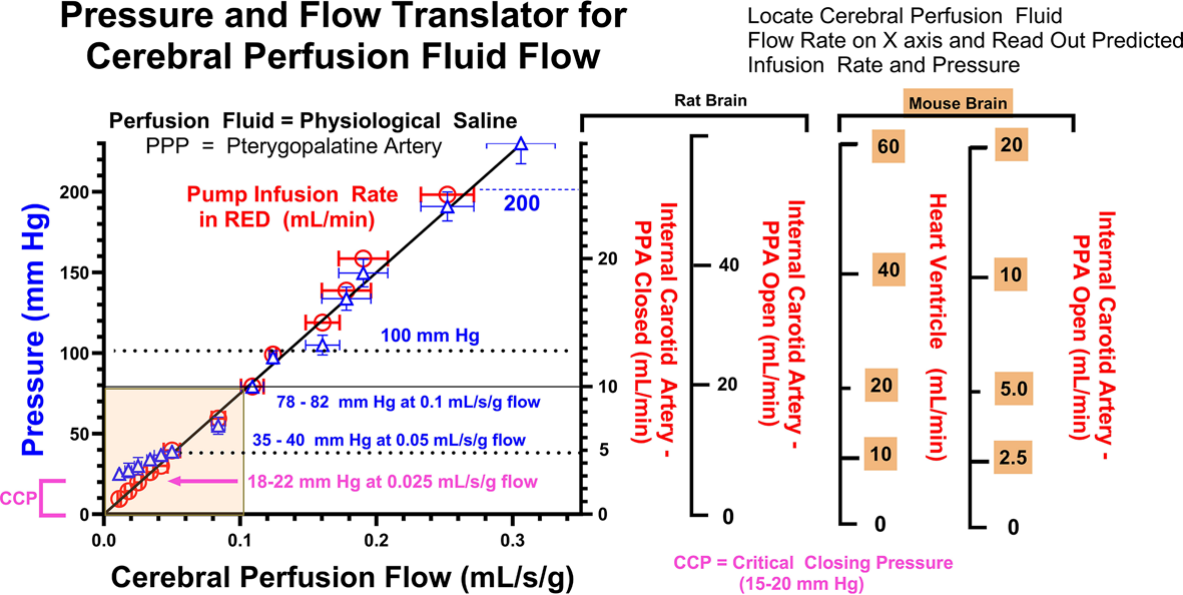
Plot of perfusion pressure and pump perfusion rate vs. measured brain cerebral perfusion flow rate. The differing pump perfusion ranges on the right are set (*left* to *right*) for 1st—Rat brain carotid artery perfusion with pterygopalatine artery closed. 2nd—Rat brain carotid artery perfusion with pterygopalatine artery open (pump values are twofold greater to obtain the same perfusion pressure). 3rd—Mouse cardiac perfusion, and 4th—Mouse brain carotid artery perfused with the pterygopalatine artery open. Plot can be used to obtain the predicted pump infusion rate desired based upon the preferred cerebral fluid flow rate and pressure.

In Fig. 16, the X axis represents pump perfusion rate (mL/min) with the left Y axis showing perfusion pressure (mm Hg) and right Y axis showing local cerebral fluid flow (cerebral cortex). Perfusion fluid is bicarbonate buffered physiologic saline fluid with no plasma protein or blood cells. Some researchers have preferred lower flow and pressure values. However, when pressure drops to <10–20% of normal, perfusions are subject to greater variability and reduced values for permeability and vascular volume, possibly because pressure levels may be inadequate to maintain vessels dependably open. Critical closing pressure in brain is 10–20 mm Hg. The Fig. 16 graph is offered to help promote greater awareness of the danger of low flow preparations and to facilitate researchers shifting to different flow rates to take advantage of the flow rates pioneered in this project. Table S1 in Supplementary Material provides a summary of flow rates reported by different groups using the in situ brain perfusion method. Papers are grouped by common techniques providing similar flow rates.

## Discussion

### High extraction brain measurement

The primary finding of this study is that cerebrovascular P_o_S can be measured with the in situ brain perfusion method for lipophilic drugs with Log P_oct_ values matching those of most FDA-approved CNS drugs. Prior to this work, most drugs in the Log P_oct_ 1-5 range were beyond the scale of in vivo BBB permeability measurement. Our results extend prior work by Fenstermacher, Rapoport, Levin and Oldendorf by ~5 orders of magnitude, with similar y-intercept and slope (0.9–1.0) (Fig. 14) [91, 92, 94, 95]. The highest measured P_o_S for a neutral solute was raised >200-fold from 0.15 to 32.1 mL/s/g for t-butyl-chlorambucil at a Log P_oct_ at 5.2. Similarly, P_o_S for the neutral species of acids and bases was taken >200-fold further to 3200–8300 mL/s/g for the long chain fatty acid, arachidonic acid and the bright dye, erythrosine B. Individual lipophilic neutral agents such as testosterone, diazepam, midazolam, estradiol and progesterone differed from the mean of previous determinations by +19, +24, +32, +52, and +194 times [28, 96–98]. The large P_o_S differences were attributed to more accurate determination of extraction differences and greater flow rates attained with the revised method. BBB P_o_S values were measured under conditions of normal BBB integrity, surface area, and vascular volume. Greater flow required validation of appropriate flow markers capable of accurately measuring flow at the elevated perfusion rates (Fig. 4). For the higher Log P_oct_ 3–6 solutes tested, the P_o_S differences were consistently 20–200-fold higher and did not represent the impact of one or two aberrant compounds. Thus, the P_o_S shift for this solute class is quite significant, and will have substantial impact, as is discussed further in this section.

### Accurate flow marker from saline at elevated flow rate

Keller and Waser [99] previously reported that brain uptake of diazepam and chlorpromazine correlate strongly with cerebral blood flow, consistent with the findings of this paper (Table 3). Pullen and Hogdson [100] showed that diazepam has a brain uptake index (BUI) significantly greater  (117%) than iodoantipyrine, the reference tracer used in many neuroscience studies. In this manuscript we show that free diazepam first pass E in brain equals or exceeds 90%, up to an incredible flow rate of 0.45 mL/s/g (27 mL/min/g) (Fig. 5). Thus, contrary to some preliminary claims, diazepam K_in_ does not underestimate saline flow to brain in the in situ brain perfusion technique. The twofold to threefold K_in_ variation observed among the drug compounds in Summerfield et al. [31], with diazepam appearing 18th in magnitude, mostly arose from the low power of the experimental design (n = 3 perfusions per compound) with 40–50 compounds. With such a design, confidence limits restrict the number of comparisons that are designated as statistically significant. We tested most of the proposed alternate agents (i.e., chlorpromazine, imipramine, sertraline, amitriptyline) and found they had a significant flow range but were not superior to diazepam. Using the Crone Renkin equation, we calculated for each tested solute the F rate that provides 90% E as P_c_S/2.3 for physiological saline in the absence of plasma protein. Of the compounds tested in this study, diazepam was 9th on the list, with arachidonic acid, palmitic acid, and midazolam being superior (Table 6). When plasma protein is present in the perfusion fluid, the priority shifts to F rate for 95% E = f_u_ × P_c_S/2.3. and diazepam appears 4th on the list with a maximal F with 95% E of only 0.05 mL/s/g (~3 mL/min/g) from rat plasma. High brain V_u,br_ is an additional positive component, allowing greater uptake time for measurement. Simply based upon E, diazepam was found to perform as predicted, working well with saline and showing more limitation from plasma, similar to many lipophilic agents. The limitation was less in rat plasma (f_u,p_ = 0.1–0.2), whereas in human plasma it was much stronger, with f_u,p_ of 0.5–1.0%.

We also performed experiments directly contrasting human and bovine albumin binding and contrasting predicted rates of intravascular binding and dissociation. Diazepam binds with significant affinity to Sudlow site II of human serum albumin with a K_A_ = 2.6 × 10^5^ M^−1^ with a dissociation rate constant, k_off_ of 0.6–0.7 s^−1^(Fig. 15) [53], which limits brain extraction from human plasma to values generally lower than observed with bovine serum albumin (Fig. 15A) or with rat plasma. Measured values for K_A_ and predicted k_off_ with human albumin favored tenfold greater intravascular equilibration than with bovine and rat albumin. This was matched with 93% brain extraction of diazepam measured with bovine serum albumin, whereas with human albumin the value was almost 4 times lower. A number of the drugs and hormones studied in this paper (e.g. chlorpromazine, paroxetine, and propranolol,) have greater k_off_ constants (2–6 s^−1^) than diazepam for human serum, predicting greater equilibration. Diazepam, with the greater binding affinity and slower off rate from human serum albumin, still performed comparably between measured E, Crone Renkin calculated E and E predicted from numerical integration given the binding constants. Thus, while the E values differed markedly between species, the differences within species for the three determinations were less, 10–20%.

### Free drug hypothesis

The data of this study lend solid support that the Crone Renkin equation can predict brain K_in_ and PS values with accuracy, as was shown for chlorpromazine, imipramine, and iofetamine in the dual method comparison of Fig. 12A and in the Fig. 11A plot of measured brain extraction vs. “f_u_ × PS/F”, showing close agreement for seven ligands and 69 different data points of flow and free fraction. This conclusion is dependent upon fulfilling the listed requirements and assumptions of the methods. These include accurate measurements of F and f_u_, perfusion fluid pH (7.4), perfusion time, tracer purity and integrity, and reasonable perfusion pressure. The combined findings from over 35 agents all suggesting a reasonable fit using the simple arterial free fraction with no need to invoke “enhanced” dissociation, is strong support for the free drug hypothesis [101]. Such was reported previously by Mandula et al. [14] for drugs that bind restrictively (E_br_ < f_u,p_) to the primary Sudlow I and II binding sites of serum albumin. Interest has continued in the field to resolve the conundrum for drugs that bind nonrestrictively (E_br_ > f_u,p_) to plasma proteins and the role of “enhanced” drug dissociation, with two major studies published recently [26, 27].

While most have suggested the problem lies in nonequilibrium conditions, we propose an alternate explanation—that researchers were simply inserting low control values (P_o_S values) in the equation, at least in some instances. The composite term in the exponential of the Crone Renkin equation is “f_u_ × P_o_S/F” for neutral solutes like diazepam. While most researchers focused on the free fraction, an equal impact is obtained by insertion of the wrong value for P_o_S—or with P_c_S for acids and bases. Use of accurately determined PS’s addresses the problem for all of the main solutes reported to give "enhanced" dissociation—chlorpromazine, imipramine, propranolol, diazepam and other benzodiazepines, testosterone, estradiol, and more. For all of these we found significantly greater P_o_S and P_c_S values.

We propose that resolution does not require steady state or equilibrium experiments. One example showing this is substitution of the carefully determined BBB P_o_S value for diazepam in the results of Jones et al. [20] and Tanaka [22], which lead the data to closely fit the Crone Renkin model (Fig. 11B, C). An additional demonstration was in the human brain and epileptic drug study of Paulson et al. [41]. They reported a brain extraction of 42% for diazepam. When we insert our new BBB P_o_S for diazepam (0.93 mL/s/g) with their extraction into the Crone Renkin equation, the resultant diazepam free faction (0.5%, see Fig. 15B) matches perfectly with values from large human data bases (0.5–1.5%).

Thus, from three separate pathways—prior brain E studies for other lipophilic drugs (imipramine, propranolol, chlorpromazine) in animals, several existing brain E studies for diazepam in animals, and the Paulson brain E study for diazepam extraction in humans, each resolves the inconsistency—with no need to invoke "enhanced" or "induced" dissociation, instead simply by inserting the new, more accurate BBB P_o_S values in the regression. This also is consistent with the fact that some drugs in prior studies actually fit the Crone Renkin equation (e.g., propranolol [18]). Propranolol was one of the agents that showed only a limited difference in P_o_S with our study

Further, Jones et al. [20], in their brain uptake index study of diazepam using fatty acid free human serum albumin, calculated that brain vascular P_o_S of 55.7 mL/s/g fit the Crone Renkin equation with no need to propose high f_u_ values ("enhanced" dissociation). When we analyzed their data, we found instead that a P_o_S value of 0.8–1.2 mL/s/g appeared fully able to fit their measured extraction data, as estimated from their graphs. Our most recent BBB P_o_S to diazepam (0.93 mL/s/g) fits well with their data, based upon our own internal calculations. We noticed that 60 times our mean BBB P_o_S value for diazepam (55.8 mL/s/g) is a very close match to the number reported in their table. Thus, we suggest that perhaps Jones et al. had a simple labeling unit error in their table where the value reported was labeled in seconds but it was actually in minutes. Thus, we believe that Jones et al. may have had it right all along.

### Linear relation between Log P_o_S and Log P_oct_

The findings of this paper also demonstrate a clear linear relation between permeability and lipophilicity, whether measured as P_oct_ or Log D_oct pH7.4_ over ~10 orders of magnitude, Research has seesawed back and forth on this topic going from the 1960’s and 70’s where some investigations found a linear relation between Log P_o_ and Log P_oct_ or Log D_oct pH7.4_, whereas others found an inverted U-shaped relation [11, 31, 102, 103]. Passive diffusion theory supports a linear relationship, where the slope is close to one, reflecting the predominance of partition in the lipid membrane in overall contribution to permeability. Multiple correlation analysis demonstrated a strong correlation (R^2^ = 0.957, df = 75) with a slope for P_o_ vs. Log P_oct_ of 0.91 ± 0.03 (*P* < 0.001), and a slope for P_o_ vs. Log MW of −0.96 + 0.17 (*P* < 0.001) over 10 log units. The 0.91 slope value for Log P_oct_ reflects the importance of solute partitioning in the lipid membrane bilayer and is quite close to the theoretical optimum of 1.0, which is a critical component in the permeability definition [6, 92, 104]. Likewise, solute diffusion in the membrane core was reported by Anderson 1999 to show a greater size dependence than that noted for the traditional Log P_o_S vs. Log P_oc__t_/MW^0.5^ plot. Thus, while adequate plots were obtained in Fig. 13 for Log P_o_S vs. Log P_oc_/MW^0.5^, literature reports indicate a greater dependence on size in lipid bilayers, Thus, the highest correlation was found vs. Log 1/MW [95, 104]. The statistically significant inverse relationship between P_o_S and molecular weight was important, as MW has been cited as a limiting factor in BBB penetration, including the Lipinski Rule of Five as adopted for the BBB. The “U-shaped” relation has been linked to reports of lower lipid solubility in aqueous medium for more lipophilic species [105].

### Comparison to permeability with other methods and tissues

Brain vascular P_o_S results in this study matched for two solutes within twofold to threefold to values obtained by regression analysis with the in vitro PAMPA database [30]. One of these was imipramine (twofold difference; our P_o_S = 21 vs. 9.8 mL/s/g for PAMPA correlation based on the reported Log P_o_ = −1.01). The other was sertraline (our value P_o_S value 31 mL/s/g vs. 12 via PAMPA correlation from Log P_o_ = −0.91 ± 0.51). On the other hand, the difference was sevenfold for amitriptyline (our P_o_S value = 76 mL/s/g vs. 10 via PAMPA correlation with Log P_o_ = −0.99 or –0.91 ± 0.51) and 20-fold for indomethacin (our P_o_S = 14 vs. 0.7 mL/s/g via PAMPA correlation with Log P_o_ = −1.01) [30]. The average difference among these four solutes was eightfold, with PAMPA coming in below in situ values. The difference may in part reflect transport by uptake carriers in situ as a large number of anion and cation transporters are known to be expressed in vivo at the BBB. While the permeability values of this paper lay silent on specific transport mechanisms, the values are important for setting the standard upon which in vitro and in silico studies can be compared to in vivo [56].

The lipophilic P_o_S values listed in Table 3 compare within an order of magnitude to values published for other organs. For example, the BBB P_o_S measured for diazepam in this work (0.90 mL/s/g) compares very reasonably to 0.4 mL/s/g reported by Chou and Rowland in 1995 for liver membrane [106], though S for liver parenchyma may be greater than S for brain capillary endothelium. Similarly, the reported palmitate P_c_ for cardiac endothelium of ~0.5 to 1 cm/s is 20-fold greater than our value of 0.03 cm/s for palmitate [107, 108].

### Perfusion fluid mechanics and flow rates

Brain vascular flow and pressure values were carefully chosen in this study to provide stable BBB PS values that can be compared to those at normal pressure and flow. Rat BBB PS demonstrated a linear relation with physiologic saline perfusion rate (Fig. 5) where control pressure was selected to match that at the circle of Willis (~80 mm Hg) [58]. With the perfusion fluid in many instances containing no cells or plasma protein, a fourfold greater perfusion pump rate was required to match the pressure of the Circle of Willis because the saline viscosity is fourfold lower than whole rat blood [93]. A number of researchers chose flow rates recommended in the original Takasato et al. paper fearing injury from high flow rate and pressure to the brain vasculature. However, as shown in this report, the measured pressure at the carotid cannula matches that of the Circle of Willis in vivo, and is comparable in shear stress because shear stress, by definition, is proportional to flow times viscosity [109]. Thus, with a flow rate fourfold elevated and the viscosity one fourth that of blood, the shear stress at the recommended flow rate actually matches that normally in vivo. Application of flow rates 50%, 25% (1/4th—matching in vivo) or 10% of that recommended would predict perfusion pressures of 40, 20 and 8 mm Hg, with the last two values unfortunately close to critical closing pressure where the consistent flow structure seen at higher rates becomes more intermittent.

### Control markers—PS

Control markers, such as antipyrine, with PS > 10^–3^ mL/s/g, should also be measured simultaneously with flow to correct for flow dependence in permeability. In this study, we measured PS for 4 different permeability markers. Two (thiourea and ethylene glycol) had PS values in the 10^–4^ range with E = 2–4%. For these solutes, flow was not rate limited and uptake K_in_ was determined by permeability. The other two tracers had uptake K_in_ values ~50-fold greater so that both flow and permeability contributed with a solid component from surface area. All four permeability markers showed PS values that were remarkably stable over an ~tenfold range in F (2.5 vs. 25 mL/min as pump rate). When data were pooled so that each flow rate value had n = 11–23 perfusions, then a trend to greater PS values was observed of ±20–30% as flow values increased toward 25 mL/min. A similar range was reported by Duelli and Kuchinsky et al. (18%) in capillary surface area with flow [90]. The trend was observed across the entire range of compounds studied (n = 125), which also was consistent with a surface area effect. In addition, it was clear that nonlinear regression was more consistent and stable in K_in_ than with the linear sloping method—particularly for compounds with greater PS values than 0.002–0.005 mL/s/g [3]. Together, these factors may account for the threefold lower K_in_ for antipyrine reported by some groups with the perfusion method [30, 31]. We found the same trend in our data when flow rate was set to a lower value (0.008–0.035 vs. our standard 0.09–0.11 mL/s/g), where, as shown in Fig. 6A, antipyrine K_in_ dipped from 0.02 to 0.006 mL/s/g. Fenstermacher, who used antipyrine widely in a number of papers in the 1990s and 2000s consistently got brain vascular P_o_S values that matched reasonably well with ours (PS = 0.02–0.025 mL/s/g). It is important to verify barrier passive permeability and surface area in any study of modified physiology or disease. The excellent agreement of our PS in situ values with those from prior compilations by Fenstermacher and Rapoport [91] (Fig. 12B) add further support to the accuracy of these measurements.

### Two compartment model

The limitation with the sloping method for curve fitting arises from difficulty in determining at which points backflux exceeds 20%, particularly when the method is used in an "explorer" mode. In contrast, the nonlinear regression method provides brain distribution volume estimates (V_u,br_) that can be employed to assess the backflux assumption (i.e. C_br_/(C_u,pf_ × V_u,br_) < 0.2). At the highest flows tested (46 mL/min), BBB permeability increased sharply for thiourea at perfusion pressures >200–240 mm Hg, as previously reported [58]. Therefore, most experiments were limited to flow rates (≤0.30 mL/s/g) where barrier to polar markers was maintained to a sufficient degree. Further, for highly lipophilic molecules traversing the barrier predominantly by transcellular mechanisms, even a tenfold to 20-fold increase in BBB paracellular permeability would be predicted to have limited effect on brain uptake for a highly lipophilic compound.

### Brain equilibration t_1/2_

As part of this study, brain P_o_S values were used to calculate the brain rate constant for solute backflux into the circulation and the t_1/2_ for brain equilibration. As shown in Fig. 14E, most CNS drugs in the Log P_oct_ 1–5 range had brain t_½_ values <100 min. Bases with higher V_u,br_ values averaged t_1/2_ of ~20 min, vs. t_1/2_ values of ~2 to 3 min for acids and neutral solutes. Prior surveys report that most FDA-approved CNS drugs are lipophilic bases (75%); with ~19% being lipophilic neutral drugs and 6% being acids. When the library of drugs was modified to match that reported in the literature, the average t_1/2_ for brain equilibration is considerably longer (30 ± 3 min) but is still shorter than t_1/2_ values provided by systemic pharmacokinetics. As noted in the figures, CNS basic drugs (n = 12) showed a pattern of free drug extraction (99 ± 0.3%) that matched that of CNS neutral drugs (100%), whereas CNS acidic drugs had a free drug extraction that was only half that of the other agents, 45 ± 9% for total E. However, lipophilic acids bound strongly to plasma proteins (mainly serum albumin) to reduce their total brain extraction to only 1.8% (see Table 6), whereas neutral and basic drugs had total extractions (~100 and 32%, respectively) that were tenfold to 50-fold greater. Thus, it is interesting that most current CNS drugs are bases or neutral drugs.

The fall off in K_in_ for acids is prominent in Fig. 13 and is consistent with findings in the field regarding drugs with lower brain availability. For 16 compounds, we compared our predicted brain equilibration t_1/2_ values to measured values and found reasonable agreement within a factor of 2–3 (Table 7). Variation, expressed as a measured/predicted ratio, ranged from 0.61 to 1.7. The average was 1.02, with SEM = 0.07 and CV = 27%. Predicted values are based upon rat plasma f_u,p_ and rat f_u,br_ or V_u,br_ values.

As recognized by Brodie, Bradbury and Fenstermacher [6, 110, 111], the BBB is not limiting to the rate of access of most CNS approved drugs that lack substantial BBB active efflux transport. Given that ~75% of CNS drugs are lipophilic bases and 19% are lipophilic nonelectrolytes, ~94% of brain drugs show 90–100% free drug extraction and thus in the absence of plasma protein, K_in_ ~ F, as shown in Fig. 15D. Consistent with this, the average K_in_ of the top 35 out of ~50 CNS drugs studied by Summerfield et al. [31] (K_in_ = 0.0319 mL/s/g) fell very close to the K_in_ value listed for the flow marker diazepam (K_in_ = 0.0329 mL/s/g). However, the predicted average K_in_ from blood can average less due to tight plasma protein binding, that shifts acids and some bases and nonelectrolytes from nonrestrictive to restrictive plasma protein binding. The critical difference here is that the numerator (f_u,p_ × P_c_S) in the exponential portion of the Crone Renkin equation must be equal to or greater than the cerebral blood flow (F) for the protein binding to be nonrestrictive. Plasma protein binding lowered the exponential composite to <1.0 for most acids and many bases, so that in the presence of normal plasma proteins, only the lipophilic nonelectrolytes as a group continued in great majority to exhibit nonrestrictive plasma protein binding effect.

Finally, new PS and *P* values are reported overall for 78 compounds for cerebrovascular permeability that are thought to have little or no component of active efflux. PS values were determined for 50 additional solutes, some of which show saturable uptake, such as by system LAT1, whereas others showed signs of marked active efflux, (paclitaxel, vinorelbine, doxorubicin). Values are presented in this study for 15 additional solutes (Table 5), reflecting approximately one third of the additional set. Their values may assist further work in that critical area.

### Application to mechanistic studies

This study shows that it is possible to obtain robust brain vascular P_o_S values for compounds that exhibit high brain extraction from saline at normal flow rates and thus likely would have been beyond the scope of accurate measurement just a few years ago. Our hope is that this work will spur researchers to transform modes of thinking and update experimental protocols to the challenges presented by this class of agents. In most cases, investigators enter this range to deliver some in vivo data to support mechanistic transport studies performed in vitro. The typical report has an in situ brain perfusion K_in_ value from saline compared to a historical F value from the same or a different laboratory. The relative extraction obtained in this manner has limited value to establishing an accurate E estimate, from our experience. In fact, it likely does more harm than good. A number of studies provide direct analyses performed in close succession, though possibly not simultaneous [80, 81]. For long chain fatty acids, rimantadine, diphenhydramine, nicotine, testosterone and estradiol, some evidence points toward K_in_/F ratios very close to 100%. We show such (E = 100%) to be definitively true for the long chain fatty acids palmitate and arachidonate when performed with flow markers simultaneously and with significant care to minimize nonselective binding. 100% extraction is also true for testosterone, estradiol, nicotine, and for a good number of the lipophilic amine drugs (amitriptyline, sertraline, chlorpromazine) tested at normal flow rates. For agents in this range, a careful extraction evaluation is an absolute requirement in our group’s experience to have a valid result. Common sense would also dictate that most all subsequent experiments must employ dual flow and pH control, given the number of compounds that are acids and bases. Further, one must remember that in common experiments to evaluate transport mechanism—i.e., (1) self-competition, (2) competition by structural analogs, (3) inhibition by defined transport blockers and (4) dependence on pH or ion concentrations, the highest caliber experiments would include dual K_in_ and flow uptake, pH internal controls, and internal controls for passive markers. Critically, if extraction is 100%, as was noted for a good number of long chain fatty acids, basic lipophilic drugs, and steroid hormones, then competitors added into the perfusion medium will also, in many instances, show similar behavior (absolutely in the case of self-inhibition), and transport analysis must take into account that average capillary concentration would vary anywhere from 50% to near 0% of perfusate concentration. The impact can be estimated as C_avg capillary_/C_arterial_ = K_in_/PS = 0.05/2.8 mL/s/g ≈ 1.7% for palmitate when studied at a flow rate of ~0.05 mL/s/g. Similar would hold, though by differing degree, to structurally related competitive analogs. Thus, for transport mechanism, a far superior picture would be presented using PS and competitor concentrations more accurately adjusted to reflect conditions in the capillary during perfusion. Our experience tells us that the methods in this paper are suited well to showing such contributions. We hope the recommendations above may be of help to researchers in this critical area.

While the main focus of the paper is on initial uptake, and not on equilibrium delivery, the results are relevant to conditions of brain imaging and acute treatment under conditions where rapidity of treatment is important. This includes status epilepticus, acute ischemia and acute excitotoxicity, where literally “time is brain,” which do not necessarily fit the model of brain distribution at equilibrium [112, 113].

## Conclusions

This manuscript makes four primary major findings: (1) BBB P_o_S can be measured in vivo for lipophilic drugs in the range of most FDA-approved CNS drugs even though most show very high brain extraction (80–99%) at normal flow rates, (2) diazepam and a number of other lipophilic CNS agents provide suitable markers for saline flow rate up to 0.4 mL/s/g with the 95% maximal flow rate determined by P_c_S/2.3 for protein-free saline and f_u,p_ × P_c_S/2.3 for plasma, (3) the Crone Renkin equation provides reasonable measure of E, K_in_ and P_o_S with the in situ perfusion technique for CNS drugs and hormones in the absence of active efflux with the issues of “enhanced” dissociation for brain likely being an artifact of control, protein-free P_o_S values which were too low in the regression analysis; we show for several prior papers that insertion of newly determined P_o_S values in the Crone Renkin equation provides a good fit to the data without the need to invoke additional factors, and (4) the P_o_S values obtained for lipophilic neutral drugs differ by 20–200-fold from previous estimates and show a linear relationship over 10 orders of magnitude between Log P_o_S and Log P_oct_ with an R^2^ of 0.94–0.95 for 78 compounds.

Molecular weight and pK_a_ are also important with the neutral fraction providing the driving force for brain entry of acids and bases. The paper also makes recommendations on use of two compartment analysis, over linear uptake, for lipophilic solutes and on the importance of dual simultaneous measurement of K_in_ and F for lipophilic solute transport into brain. Principal in this area is ensuring control over nonspecific binding as that frequently causes overestimation of the true solute exposure to brain and thus underestimation of BBB K_in_ and E.

### Recommendations

To aid in the design of quality experiments and accurate analyses, we provide the following recommendations, based on the findings reported in this paper.

### Flow rate, pressure and nonspecific binding

1. Brain perfusion rates should be set so as to maintain cerebral flow rates as well as prefusion pressure and shear stress. In our opinion, carotid perfusion pressure should be at least 40–50 mm Hg, and preferably higher (60 and 90 mm Hg), to ensure stable flow rate and pressure. Low  perfusion pressures predispose tissues to variable flow and vascular collapse. In this paper, we have found that rates <0.01 mL/s/g (resulting in <10 mm Hg pressure) lead to abnormally low P_o_S and V_v_ values. Also, excessive flow rates causing pressures >200 mm Hg can damage the BBB and predispose the preparation to changes in vascular P_o_S and V_v_ related to hypertensive injury.
2. *Brief vascular pre- and post-washes with drug-free physiological saline are recommended*. Pre-wash (20–30 s) can remove high affinity albumin binding sites from brain vascular space for compounds with high plasma protein binding (> 99%). Post-vascular wash (5–15 s) helps remove loosely bound drug absorbed to the vascular wall, as illustrated in Fig. 2C for palmitate. One compound that displays substantial nonspecific binding to the brain vascular endothelium is the thyroid hormone, l-T3. In the absence of plasma protein, l-T3 binds rapidly in 5–20 s reaching values that exceed transvascular transport by 2–3. Other compounds that display large components of nonspecific vascular binding include long chain fatty acids and cationic choline.
3. *Great care should be exercised to ensure proper maintenance of the body temperature of the test animal and of the pH and temperature of the perfusion medium*. We use a circulating water batch that surrounds the perfusion syringes with copper tubing bearing water that is heated to 37.5–38 °C. Further, all perfusion containers are closed to prevent loss of volatile carbon dioxide gas from perfusion fluid, which would result in alkaline shift of the perfusion medium.
4. *Thorough steps must be taken to screen and correct for nonspecific binding, both within the perfusion system and at the brain capillary membrane*. Solid effort at the start to establish and validate an appropriate perfusion protocol could save months of back correction experiments or the necessity to start over mid-way through an experiment.

### Drug uptake and compartment analyses and accurate determination of K_in_ and F

1. Care should be taken in all analyses to ensure that *analytical assays have the accuracy and stability to provide clear answers*. The following are critical: (a) appropriate internal controls and blanks; (b) attention to ensure binding sites are not overwhelmed and saturated with plasma protein samples; and (c) accurate sampling times, particularly at low inflow perfusion rates where delay times for perfusion fluid to reach brain capillaries can rise to 7–10 s. For *radiotracers, chromatography is critical for both perfusion fluid and brain*.
2. *For more permeant species, the two compartment model is attractive to avoid complications from increasing backflux*. However, with knowledge of the brain distribution volume, calculations can reveal the time over which uptake is linear. In these cases, the Y intercept V_o_ value is critically valuable to gain insight on the quality of the estimate. Backflux error is to be suspected when V_o_ values exceed predicted values by twofold or greater. Preferably, best fit of K_in_ and V_u,__br_ by nonlinear least squares regression using the full time course of brain uptake and equilibration can be determined. In many instances, equilibration can be achieved for free drug in <20 min.

### Protein perfusion and free drug analysis—critical considerations

1. Bovine serum albumin (not fatty acid free) was utilized in focused experiments of this study to measure brain vascular PS in the presence of plasma protein in rats and mice. *Low affinity albumin was essential to meet the assumption of the Crone-Renkin equation that drug equilibration in the vascular space was rapid.* Dissociation can prove limiting for Sudlow site I and II drugs, like warfarin and diazepam, respectively that bind with substantial affinity to human serum albumin in the absence of plasma protein. Rat albumin also has a lower affinity and likely meets the dissociation half time requirement for equilibrium during drug passage from the arterial to the venous end of the capillary bed. *In some cases, rat or human serum can serve to provide the complete mixture that exists in vivo*.
2. *Brain uptake K*_*in*_* and t*_*1/2*_* for equilibration should be measured under conditions that match those for which the experiment is intended*. For uptake comparison to rat blood or plasma, then rat serum albumin is appropriate. Similarly, for comparison to human blood, human serum albumin is appropriate, because important species differences in affinity are known. The brain K_in_ and t_1/2_ values presented in Table 5 are designed for rat blood.
3. Further, *one must balance brain K*_*in*_* and t*_*1/2*_* determinations for the impact of other parameters that are normally present in systemic blood*. For example, the diazepam free fraction in the presence of human serum albumin is known to be significantly affected by (a) the presence of chloride and calcium, and (b) circulating levels of plasma fatty acids and the amino acid, l-tryptophan, which compete with diazepam for binding to the Sudlow site II of albumin. Care must be taken to control for each of these factors so the free fraction levels match those seen under appropriate conditions in vivo*.*
4. The most rigorous and scientifically valid approach is to *start with a commercial and pharmaceutical preparation of carefully purified albumin free of fatty acid and other contaminants. To this, adjustments may be made to add in critical factors such as fatty acids and l-tryptophan that are found in circulating blood.* This latter approach also allows one to see the effect of elevated concentration of endogenous factors, such as unesterified fatty acids, which are released in greater quantities in response to heparin administration. Thus, the diazepam fraction is known to vary over a considerable range in vivo, from 10 to 20% unbound in rat blood and from 0.5 to 1.5% unbound in human blood. This is also matched by species-specific difference in albumin that make the affinity for diazepam 7–10 times higher in humans, than in bovine or in rat albumin.

## Supplementary Information

Additional file 1.

## Data Availability

The data supporting the findings of this study are available within the paper and its Supplementary Information files. Should any raw data files be needed in another format they are available from the corresponding author upon reasonable request.

## Abbreviations

AAG: Alpha-1 acid glycoprotein
BBB: Blood-brain barrier
C_a_: Arterial concentration
C_avg, cap_: Average capillary concentration
C_u_: Unbound (free) concentration
C_tot_: Total concentration (bound + unbound)
C_v_: Venous concentration
df: Degrees of freedom
D_oct pH 7.4_: Octanol/water or saline partition coefficient at pH 7.4
E: Extraction
E_free_: Extraction of free drug
E_total_: Extraction of drug incorporating plasma protein binding
F: Perfusion fluid flow
f_n_: Neutral fraction
f_u_: Unbound fraction
f_u,p_: Unbound fraction in plasma
K_in_: Unidirectional uptake transfer constant
k_off_: Rate constant for dissociation from protein
k_out_: Rate constant for efflux from brain
MW: Molecular weight
PAMPA: Parallel artificial membrane permeability assay
P_o_ or P_o_S: Intrinsic permeability or permeability-surface area product to neutral solute
P_c_ or P_c_S: Cellular permeability or permeability-surface area product to solute including all species (i.e., neutral, charged) unbound in the solution of measurement
P_oct_: Octanol/water or saline partition coefficient
PPA: Pterygopalatine artery
S: Surface area
t_1/2_: Brain equilibration half-time
V_u,br_: Brain unbound volume of distribution
V_v_: Vascular volume
